# Impacts of *Mycoplasma agalactiae* restriction-modification systems on pan-epigenome dynamics and genome plasticity

**DOI:** 10.1099/mgen.0.000829

**Published:** 2022-05-16

**Authors:** Emilie Dordet-Frisoni, Céline Vandecasteele, Rachel Contarin, Eveline Sagné, Eric Baranowski, Christophe Klopp, Laurent-Xavier Nouvel, Christine Citti

**Affiliations:** ^1^​ IHAP, Université de Toulouse, INRAE, ENVT, Toulouse, France; ^2^​ INRAE, US 1426, Get-PlaGe, GenoToul, Castanet-Tolosan, France; ^3^​ INRAE, UR875 MIAT, Sigenae, BioInfo Genotoul, BioInfoMics, F-31326 Auzeville, France; ^†^​ Present address: INTHERES, Université de Toulouse, INRAE, ENVT, Toulouse, France

**Keywords:** Mycoplasma, methylome, epigenome, restriction-modification system, horizontal gene transfer, DNA methylation

## Abstract

DNA methylations play an important role in the biology of bacteria. Often associated with restriction modification (RM) systems, they are important drivers of bacterial evolution interfering in horizontal gene transfer events by providing a defence against foreign DNA invasion or by favouring genetic transfer through production of recombinogenic DNA ends. Little is known regarding the methylome of the *Mycoplasma* genus, which encompasses several pathogenic species with small genomes. Here, genome-wide detection of DNA methylations was conducted using single molecule real-time (SMRT) and bisulphite sequencing in several strains of *Mycoplasma agalactiae *, an important ruminant pathogen and a model organism. Combined with whole-genome analysis, this allowed the identification of 19 methylated motifs associated with three orphan methyltransferases (MTases) and eight RM systems. All systems had a homolog in at least one phylogenetically distinct *Mycoplasma* spp. Our study also revealed that several superimposed genetic events may participate in the *M. agalactiae* dynamic epigenomic landscape. These included (i) DNA shuffling and frameshift mutations that affect the MTase and restriction endonuclease content of a clonal population and (ii) gene duplication, erosion, and horizontal transfer that modulate MTase and RM repertoires of the species. Some of these systems were experimentally shown to play a major role in mycoplasma conjugative, horizontal DNA transfer. While the versatility of DNA methylation may contribute to regulating essential biological functions at cell and population levels, RM systems may be key in mycoplasma genome evolution and adaptation by controlling horizontal gene transfers.

## Data Summary

The authors confirm all supporting data and protocols have been provided within the article or through supplementary data files (Figs S1–S4, Tables S1–S10) available in the online version of this article. Raw data and assembled genomes generated for this study are available from the NCBI Sequence Read Archive (SRA) and GenBank (Bioproject PRJNA717738). The complete genomes of the two reference strains used in this study, 5632 and PG2, are available in the GenBank database at accession numbers GCA_000089865.1 and GCA_000063605.1 respectively.

**Impact Statement**

DNA methylation is pivotal in the biology, adaptation, virulence, and evolution of bacteria, mainly by modulating gene expression and by interfering with horizontal gene transfer (HGT). Methyltransferases (MTase, M in RM system), often in association with cognate restriction enzymes (R), are encoded by gene repertoires, which vary across bacterial species and strains. Yet, little is known regarding the methylome of the *Mycoplasma* genus, a large group of bacteria encompassing several pathogenic species with small genomes. This study provides the first comprehensive insight into the impacts of RM systems on pan-epigenome dynamics and genome plasticity in *Mycoplasma agalactiae *, an important ruminant pathogen and a model organism. In this species, several independent but superimposed genetic events affect the expression of RM systems and are responsible for modulating the landscape of the mycoplasma epigenome, within and among strains. Most importantly, some of these systems were experimentally shown to play a major role in mycoplasma HGT, and, in particular, in mycoplasma conjugative chromosomal transfer (MCT). MCT has been shown to drive the transfer of any part of the genome, including virulence genes as well as mutations responsible for antibiotic resistance. By controlling gene flow among cells, some RM systems may be key players in mycoplasma genome evolution and host-adaptability.

## Introduction

*Mycoplasma* spp. represent a large group of bacteria that have adopted a parasitic lifestyle and closely interact with their human or animal hosts, as commensals or pathogens. These organisms belong to the Mollicutes class and encompass some of the simplest life forms capable of self-replicating under laboratory conditions in axenic media. From a phylogenetic point of view, mycoplasmas derived from a common ancestor to Gram-positive bacteria with a low GC content and followed an evolutionary scenario often described as a ‘degenerative evolution’ because of successive and drastic genetic losses. As a result, current mycoplasmas are characterised by a small genome (ca. 0.5 to 1.35 Mbp), no cell wall or cell wall precursors, and a restricted number of metabolic pathways (for a review see [1, 2]). For decades, genome reduction has been proposed as the only force driving mycoplasma evolution. Horizontal gene transfer (HGT) was then considered marginal in these organisms because of their limited content in efficient recombination systems [3] and their paucity in mobile genetic elements (MGEs) [4, 5]. A paradigm shift occurred within the last 20 years with the first discoveries of conjugative MGEs in mycoplasmas [6, 7] and large chromosomal DNA exchanges between phylogenetically remote *Mycoplasma* spp. sharing the same habitat [5, 8, 9]. *In vitro* experiments further demonstrated that mycoplasmas harbouring integrative and conjugative elements (ICEs) have retained a form of sexual competence [10, 11]. Most specifically, our group highlighted two conjugative processes occurring within and among strains of *M. agalactiae *, an important pathogen of ruminants and a model organism. The first was the conventional, horizontal dissemination of mycoplasma ICEs (MICEs), from ICE-positive to ICE-negative mycoplasma cells [10, 12]. The second involved the transfer of chromosomal DNA [11, 13], a conjugative process initially described from ICE-negative to ICE-positive cells and further designated as MCT for mycoplasma chromosomal transfer [14]. MCT is an atypical mechanism of HGT that is not physically linked to MGE movements but relies on the MICE conjugative machinery. For MCT to occur, one mating partner must carry a functional MICE [10, 12]. The mechanism driving MCT remains to be fully understood, but its outcome has been well described. Multiple, large, and small chromosomal regions of the recipient cell are simultaneously replaced by the donor-counterparts via recombination, as described for the distributive conjugative transfer (DCT) in Mycobacteria [15, 16]. While the frequency of such a phenomenon is low, the impact of MCT has far-reaching consequences, as the mating of two strains is able to generate progenies composed of a variety of individual mosaic genomes, each being unique [13, 14]. One recurrent observation was the apparent polarity of the chromosomal transfer during mating experiments involving *M. agalactiae* strain 5632, with this particular strain always being identified as the recipient genome regardless of its mating partner. The same was observed when conjugation was bypassed by PEG-cellular fusion, suggesting that the asymmetry of the chromosomal transfer might be independent of the conjugative mechanism itself [14].

Restriction modification (RM) systems are key players of bacterial HGT by protecting their host genome from incoming MGEs or by promoting recombination of the invading DNA, as recently suggested [17, 18]. Furthermore, detailed analyses suggest that the small genome of strain 5632 has a relatively large arsenal of RM systems [19], raising the question of their role in the apparent polarity of MCTs.

RM systems are classically divided in three categories depending on their composition, sequence recognition and cleavage position (for a review see [20, 21]). Briefly, Type I RM systems are the more sophisticated with modification and specificity subunits being both required for modification. This multi-subunit complex targets bipartite specific sequences interspaced by a fixed number of nonspecific nucleotides and once combined to the restriction subunit, cleaves up to several kilobases away from the non-methylated motif site. Type II are the simplest with a distinct methyltransferase (MTase) and restriction enzymes (REase) that respectively target short palindromic motifs, with cleavage occurring close to or within the non-methylated motif sites. Finally, in Type III RM systems the MTase is composed of multiple modification subunits that methylate short, non-palindromic sequences. Non-methylated motif sites are targeted by a separate restriction enzyme which is associated to the MTase and cut ca. 25 bp from the motif. Regardless of the RM system, MTases induce three types of base methylations: *N*6-methyladenine (m6A), *C*5-methylcytosine (m5C), and *N*4-methylcytosine (m4C), the latter being only found in Bacteria and Archaea. Most of these methylated bases are induced by DNA adenine MTase (Dam) or DNA cytosine methyltransferase (Dcm) encoded by specific *dam* and *dcm* genes. Besides protecting genomic DNA from cleavage and degradation by cognate REases, MTases also contribute to several bacterial processes, such as DNA mismatch repair, gene regulation, replication initiation, cell cycle progression, and phase variation [22]. The small mycoplasma genome lacks most known transcription factors and regulatory pathways. Despite this limitation, mycoplasmas can respond to environmental stress and metabolic insults [23–25], suggesting other types of regulation, such as DNA methylation, as epigenetic modulators of gene expression.

The Tenericutes phylum corresponds to the class Mollicutes and has a high density of RM systems, an interesting observation for these peculiar bacteria with reduced genomes [17]. In this phylum, RM systems are ubiquitous and found in 74.2 % of the genomes. Their distribution patterns are very diverse, with more than 2040 MTases in the Restriction Enzyme database (REBASE) that were predicted from 387 sequenced mycoplasma genomes [26], and an average of five predicted MTases per genome. While this highlights the importance of MTases and RM systems in mycoplasmas, their role in cellular regulation and HGT has yet to be addressed. Genome methylation in bacteria is an area of great interest because of its broad implications for evolution, biology, and virulence [27]. The development of real-time single molecule sequencing (SMRT-seq) has allowed the detection of methylated bases, whether on a plasmid or bacterial chromosome, and is particularly suited to small mycoplasma genomes. However, the characterisation of Mycoplasma epigenomes at a single-base resolution is still scarce, with only two comprehensive studies. The first one was conducted in 2013 with the type strains of two human *Mycoplasma* spp. [28]*, M. genitalium* and *M. pneumoniae *. This study revealed two different m6A methylated motifs, one common to both bacteria (5′-CTA^m6^T-3′) and one specific to *M. pneumoniae* that corresponded to the Type I methylated motif (5′-CT(N)_7_A^m6^TR-3′). Functional and distribution analyses further suggested a potential regulatory role for these methylations in cell cycle and gene expression. More recently, the *M. bovis* reference strain PG45 was included in a large-scale epigenetic analysis comprising more than 200 bacterial and archaeal genomes, which were analysed by SMRT-seq [29]; five different methylated motifs, four with m6A and one with m4C, were identified for 14 putative MTases identified in the PG45 genome. While providing the first insights into the mycoplasma epigenome, these studies were limited to PacBio SMRT-seq, which does not allow the detection of m5C modifications, and to a single strain per species.

The current study provides new insights into the extent, variability, and impacts on gene transfers of DNA methylation in *M. agalactiae *, a model species for the understanding of mycoplasma HGTs. This was achieved by combining SMRT-seq and Illumina bisulphite sequencing (BS-seq) for the detection of all cytosine modifications (m4C and m5C) in addition to m6A. In-depth analysis of the *M. agalactiae* epigenome landscape was conducted using two reference strains, namely 5632 and PG2, for which circularised genomes were available, as well as data on HGT and genetic tools. Eight additional field strains, with varied histories, were also used to assess the methylome diversity within the *M. agalactiae* species and identify the repertoire of active RM systems along their recognition motifs. This knowledge was then used to address the impact of RM systems on HGTs by experimentally demonstrating the influence of DNA methylation on the outcome of mycoplasma conjugation and by establishing a correlation between mobilomes and active RM repertoires of *M. agalactiae* genomes. MTases identified in this study all have homologs in at least one other *Mycoplasma* spp. of a different phylogenetic group, indicating that data obtained with *M. agalactiae* can be extrapolated to several other Mollicutes. Altogether, our data highlight the intra-clonal and intra-species versatility of the *M. agalactiae* epigenome and unveil the dual role played by RM systems in HGT. While some RM systems provide the mycoplasma with an ‘immune system’ to prevent foreign DNA from entering, others may play a major role in HGT by promoting the incorporation of the donor chromosomal DNA into the host genome. Finally, the over-representation of RM systems in these minute-size bacterial genomes [17] might be directly linked to their role in mycoplasma host adaptation and evolution.

## Methods

### Mycoplasma strains, culture conditions, and DNA extraction

The *Mycoplasma agalactiae* strains used in this study are described in Table 1. The 5632 H1-2 [14] and the PG2-ICEA+ [10] variants were previously generated and derived from strain 5632 and PG2, respectively. Briefly, the 5632 H1-2 variant is a hybrid obtained by PEG-cellular fusion of 5632 with PG2 and is characterised by the 5632 genomic-background in which the *hsd* locus has been replaced by its PG2 counterpart, in addition to four other unrelated regions [14]. PG2-ICEA+ is a PG2 variant having acquired and integrated a functional ICEA_5632_ after conjugation with 5632. All strains and variants were grown for 24 to 48 h at 37 °C in SP4 medium supplemented with cephalexin (500 µg ml^−1^) and were cloned by serial passages in broth and solid media of a single colony. Mycoplasma cultures were stored at −80 °C.

**Table 1.** Features of *Mycoplasma agalactiae* strains used in this study

Strains	Host	Tissue sample (Associated symptoms)	Date of isolation	Geographical origin (French departments)	ST	Estimated genome size (bp)	no. of contigs	%GC	N50 value	no. of CDS
**5632**	Caprine	Joint	1991	Spain	ST-13	1 006 702	1 circularised	29.7		752
**PG2**	Caprine	Unknown	1952	Spain	ST-06	877 438	1 circularised	29.62		826
**4021**	Ovine	Milk (asymptomatic)	1988	France (12)	ST-10	887 230	3	29.7	822 961	745
**4025**	Ovine	Milk (asymptomatic)	1988	France (12)	ST-11	887 230	3	29.7	822 961	874
**4055**	Caprine	Milk (mastitis)	1987	France	ST-03	926 904	1	28.6	926 904	811
**4210**	Ovine	Milk (mastitis)	1982	France (73)	ST-05	884 408	4	29.7	614 215	719
**5276**	Ovine	Milk (mastitis)	1977	France	ST-12	890 157	3	29.7	781 215	737
13377	Caprine	Ear	2003	France (79)	ST-08	1 266 155	20	29.6	115 104	892
14634	Ovine	Milk (mastitis)	2006	France (64)	ST-12	905 745	6	29.7	615 197	751
14668	Caprine	Lung (pneumonia)	2006	France (7)	ST-14	967 428	4	29.8	808 260	811

*CDS identified by the NCBI Prokaryotic Genome Annotation Pipeline (PGAP).

ST: Shared type determined for European *M. agalactiae* strains in Nouvel *et al*. (2012) [66].

CDS: Coding DNA sequence N50 value: shortest contig length needed to cover 50% of the genome.

Genomic DNA was extracted from mycoplasma cells after 48 h of growth (beginning of stationary phase) using the phenol-chloroform method as previously described [30]. To assess the methylation status, depending on the growth phase of *M. agalactiae *, chromosomal DNA of 5632 was also extracted after 24 h, during the exponential growth phase.

### SMRT, BS-seq, and sequence annotation

Genome sequencing of *M. agalactiae* strains was performed at the GeT-PlaGe Genotoul facility (Toulouse, France) using Pacific Biosciences SMRT RSII technology (PacBio, Pacific Bioscience, CA, USA). The SMRTbell library was constructed by pooling 20 barcoded samples (ten were used in this study). Barcoded library preparation and sequencing were performed according to the manufacturer’s instructions ‘Preparing SMRTbell Libraries using PacBio Barcoded Adapters for Multiplex SMRT Sequencing’ (Pacific Bioscience). Genomic DNA was quantified at each step using the Qubit dsDNA HS Assay Kit (LifeTechnologies, France) and a Qubit Fluorometer (ThermoFischer Scientific, MA, USA). DNA purity was tested using a Nanodrop (Thermofisher Scientific). Size distribution and degradation were assessed using a fragment analyser (AATI) High Sensitivity NGS Fragment Analysis Kit. The DNA of each sample was sheared at 9 kb using the Megaruptor system (Diagenode, Belgium) and then barcoded using the SMRTbell Barcoded Adapter Prep Kit (Pacific Bioscience). Briefly, each sample underwent one-step end repair, and the barcode adapters were ligated. The barcoded samples were then pooled in equal amounts to obtain a final pool, which was purified with AMPure PB beads (Pacific Bioscience) at ×0.45 and quantified. This pool then underwent DNA damage repair and exonuclease digestion and was purified with AMPure PB beads at ×0.45. A size selection step using a 6 kb cut-off was performed on the Blue Pippin Size Selection system (Sage Science, MA, USA) with the 0.75 % agarose cassettes, using Marker S1 high Pass 6–10 kb V3 (Sage Science). The sized pool was purified again with AMPure PB beads at ×0.45 and quantified. Conditioned Sequencing Primer V2 was annealed to the size selected SMRTCell library. The annealed library was then loaded on four SMRTCells using P6–C4 chemistry on the PacBio RSII sequencer with 360 min movies. Sequencing reads were *de novo* assembled using the Hierarchical Genome Assembly Process (HGAP) protocol RS_Assembly v.3 implemented in SMRT Analysis software v.2.3 with default parameters (https://smrt-analysis.readthedocs.io/en/latest/SMRT-Pipe-Reference-Guide-v2.3.0/). An average of 1×10^5^ reads per strain were obtained, corresponding to an average of 100X coverage.

Open reading frame (ORF) prediction and automatic annotation were performed using Rapid Annotation using Subsystem Technology (RAST) [31]. GenBank editing and manual expert inspection were performed using Artemis 16.0.0 [32] and ACT 13.0.0 0 [33].

### Methylome analysis (SMRT and BS sequencing)

Methylome analysis was performed using a combination of PacBio SMRT-seq, BS-seq, and comparative genome analysis. With the SMRT data, epigenetic modification at each nucleotide position was determined based on the kinetic variations (KVs) in the nucleotide incorporation rates, and methylated motifs were deduced from the KV data. SMRT-seq only identified m6A and m4C modifications. Base modifications and detection of methylated motifs were performed with SMRT analysis software (SMRT-v2.3.0) following *de novo* genome assembly using the ‘RS_Modification_and_Motif_Analysis.1’ protocol using a QV threshold of 60. A QV of 30 was also tested, but a QV of 60 was retained because it allowed the detection of *N*4-methylcytosine (m4C) in addition to *N*6-methyladenine (m6A). SMRT methylation motifs with a mean score of up to 40 (corresponding to a *P*-value of 0.0001) were considered specific and were used for further analysis (https://www.pacb.com/wp-content/uploads/SMRT-Tools-Reference-Guide-v8.0.pdf). The SMRTView browser (v2.3.0) was used for visualisation and analysis of SMRT-detected methylated motifs. Notably, a bias was observed at each end of the contigs, with several sites not being detected as methylated within the first two and last two kbps. This was due to an intrinsic characteristic of SMRT-seq, whose coverage across a genome closely follows a Poisson distribution, resulting in a coverage decrease at each end of the contig. When the coverage in these areas was less than 25X per strand, the detection of methylated sites was not possible. This was, however, circumvented by comparing the 5632 and PG2 methylomes. Counterparts of contig extremities in one genome were 100 % methylated in the other and vice versa, confirming the technical detection bias. This was possible because the organisation and sequence of the two genomes were very similar.

The 5632 methylome was evaluated after 24 or 48 h of growth to define the impact of the growth phase on the methylation status. SMRT analysis detected the same methylated motifs in the exponential phase (24 h) and the stationary phase (48 h), with a decrease of approximately 20 % of the m6A methylated sites at 24 h and 40 % for m4C (data not shown).

Illumina sequencing of bisulphite-treated chromosomal DNA (termed BS-seq) was performed to reliably detect 5-methylcytosine (m5C). Two reference strains 5632 and PG2 were used for BS-seq, and the 5632-like strains 13377, 4025, 14668, and 4055 were chosen based on Dcm encoding gene detection by blast analyses against the Type II m5C methyltransferase genes databases (dcm database) available on REBASE (Table S1) [26]. BS-seq was performed at Novogene (UK) and included DNA fragmentation into 200–400 bp using Covaris S220, DNA libraries, and bisulphite treatments (EZ DNA Methylation Gold Kit, Zymo Research, CA, USA). Libraries were sequenced using a HiSeq 2500 platform, which enables paired-end sequencing and generates 125 bp long reads. The BS-seq raw data sets were analysed with Bismark software (v0.22.1) to detect m5C modifications and identify corresponding methylation motifs. Raw reads were mapped on the finished reference genomes using Bismark Bisulfite mapper v0.22.1 (https://www.bioinformatics.babraham.ac.uk/projects/bismark/) in non-directional mode. The methylated cytosines were extracted with bismark_methylation_extractor using --comprehensive, --merge_non_CpG, and --CX options. BedGraph and cytosine_report outputs were generated. The sequence context was analysed by extracting ±10 bp around each identified methylated cytosine. The MEME-ChIP suite (Motif Analysis of Large Nucleotide Datasets, v5.1.0; http://meme-suite.org/) was used to determine a context consensus at methylated positions, and Artemis 16.0.0 [32] was used for visual inspection.

RM system-encoding genes and methylation motif(s) co-occurrence were addressed by comparative genome analysis. All identified ORFs were searched for similarity to known RM systems using BLASTP and the 5632 and PG2 reference genomes and the REBASE database (http://rebase.neb.com/rebase/rebase.html) [26]. Significant BLASTP hits were selected using a cut-off e-value of <0.001 and exhibiting over 30 % similarity across at least 80 % of the sequence length. The active MTases were also analysed by BLASTP on NCBI (https://blast.ncbi.nlm.nih.gov) to determine their occurrence within Mollicutes and other bacterial classes. Significant BLASTP hits were selected using a cut-off e-value of <0.001 and exhibiting over 50 % similarity across at least 50 % of the sequence length.

### Sanger sequencing and fragment length analysis to define poly(GA) and poly(G) lengths

During *de novo* genome assembly, repetitive sequences can lead to erroneous rearrangements, deletions, and collapsed repeats, even with long read sequencing data. To identify the most representative number of (GA) and (G) and the variability of these repeat tracts in Type III and CpG MTases sequences, respectively, PCR was performed with primers described in Table S2 according to the Phusion High-Fidelity DNA polymerase supplier recommendations (New England Biolabs, MA, USA). PCR products were then subjected to Sanger DNA sequencing and fragment length analysis (FLA) using primers 1F_typeIII and 1R_CpG (Table S2) by Eurofins Genomics (Ebersberg, Germany). For FLA, primers were labelled with 6-carboxyfluorescein (6-FAM) in 5′.

### Transformation and complementation of *M. agalactiae* strains

Six MTase genes of 5632 (MAGa1570, MAGa1580, MAGa2700, MAGa3950, MAGa4250, and CDSH) were independently cloned into PG2, a strain devoid of these genes or having no active form (Table 2). As previously described, each of the six genes was placed under the control of the constitutive *M. agalactiae* P40 promoter [34] and inserted at the *Not*I restriction site of a mini-transposon. This mini-transposon is derived from the Tn*4001* transposon, contains a gentamicin resistance gene (*aap*), and is located in the pMT85Gm, a plasmid that cannot replicate in mycoplasmas [35]. Thus, complementation was obtained by inserting the mini-transposon and the MTase of interest in the genome. This construction has the advantage of (i) being stable because the transposase encoded by the non-replicative pMT85Gm is placed outside the mini-transposon and is lost after integration of the mini-transposon and (ii) preventing potential lethal effects due to enzyme overexpression when carried on a replicative plasmid.

**Table 2.** DNA methyltransferases detected by REBASE database in 5632 and PG2 genomes

5632	PG2	Product	MTase MS 5632 /PG2	Predicted Motif REBASE	Predicted Methylation	REase	REase MS 5632 /PG2
**Type I**							
MAGa6290	MAG5650	HsdM	+ / -	None	m6A	+	+ / -
MAGa6350	MAG5730	HsdM	+ / +	None	m6A	+	+ / -
**Type II**							
MAGa2700	*MAG2550 ^#^*		- / -	GATC	m6A	+	- / -
MAGa3950	No homolog	Dcm	+/ na	GGNCC	m5C	+	- / -
MAG**a**4250	No homolog	Bsp6IM	+ /na	GCNGC	m5C	+	+ / -
*MAGa4470 ^#^*	*MAG4250 ^#^*	Pseudogene of CPG methyltransferase	- / -	CG	m5C	−	na
*MAGa4480 ^#^*	*MAG4260 ^#^*		- / -			−	na
MAGa7650	MAG6680		+ / -	GANTC	m6A	−	na
No homolog	MAG3310	Cytosine Methyltransferase	na / -	None	None	−	na
No homolog	MAG4030		na / -	None	m5C	−	na
MAGa3200/MAGa5050/MAGa6900	No homolog	CDSH (ICEAI, II and III)†	- / na	None	None	−	na
**Type III**							
MAGa1570	MAG1530‡		+ / +	None	None	+	+/+
MAGa1580	MAG1530‡		+ / +	None	None	+	+/+

*This table is a compilation of Nouvel *et al.* [18] and this study.

†The corresponding encoding gene is present on each of the three ICEA copies present in the 5632.

‡MAG1530 homologs are present in two copies in the 5632 genome (MAGa1570 and MAGa1580, see Fig. 4).

MS, detected with tandem mass spectrometry; MTase, methyltransferase; REase, restriction endonuclease; #Italic, pseudogenes; na, not applicable; None, not predicted by REBASE.

*M. agalactiae* transformation was performed as previously described, with some minor modifications [36]. Briefly, a culture containing 10^8^ c.f.u. ml^−1^ was centrifuged at 10 000 ***g*** at 4 °C for 20 min. The pellet was washed twice with sterile cold DPBS (Dulbecco’s phosphate-buffered saline, Sigma-Aldrich, MO, USA) 1X and centrifuged at 10 000 ***g*** at 4 °C for 10 min. After centrifugation, cells were resuspended in 375 µl of cold 0.1 M CaCl_2_ and incubated on ice for 30 min. Cold CaCl_2_-incubated cells (100 µl) were gently mixed with 10 µg of yeast tRNA (Life Technologies, CA, USA) and 3 µg of pMT85Gm MTase gene-harbouring plasmids or the pMT85Pur plasmid, which carries a puromycin resistance *pac* gene (GenBank: ACR82772.1). One millilitre of 50 % PEG 8000 (Sigma-Aldrich) was then applied for 1 min, and the reaction was stopped by the addition of 5 ml SP4 liquid medium. The cells were incubated for 3 h at 37 °C, and the transformation mix was centrifuged at 8000 ***g*** at room temperature for 8 min. The pellet was suspended in 1 ml SP4 liquid medium and 300 µl were plated onto selective SP4 solid medium supplemented with 50 µg ml^−1^ gentamicin or 5 µg ml^−1^ puromycin (Sigma-Aldrich) and incubated at 37 °C for 3 to 5 days. Colonies obtained on selective solid media were picked and transferred into 1 ml SP4 liquid medium supplemented with gentamicin (50 µg ml^−1^), and incubated at 37 °C for 24 to 96 h. Transformants were stored at −80 °C.

To demonstrate the association between MTases and cognate methylated motifs and to validate the complementation efficiency, restriction assays were conducted in parallel. For this purpose, chromosomal DNA, extracted from PG2 and complemented PG2 clones using the phenol-chloroform method [30], was subjected to commercially available enzymes *Sau*96I, *Fnu4*HI, *Dpn*I, and *Dpn*II according to the supplier’s recommendations (New England Biolabs) and analysed by agarose gel electrophoresis 1 % TAE 0.5X (Fig. S1). For the other MTases, there was no available commercial enzyme that corresponded to the recognition site, and for one, CDSH, the corresponding methylation site was not known.

### Conjugation experiments with PG2 clones complemented with 5632 MTases

Mating experiments were conducted as previously described [10] using the 5632TH3 tetracycline-resistant clone [14] as the recipient strain and, for each complemented MTase, a pool of ten PG2 clones carrying a single copy of the 5632 MTase gene inserted into the chromosome. This strategy was used to avoid potential negative effects linked to mini-transposon insertion that occurred at random and might affect growth or conjugation. The mating efficiency was determined as the number of transconjugants divided by the total CFUs and compared to a control performed with 5632TH3 and a pool of ten PG2 clones having the non-complemented Gm^R^ transposon integrated at different chromosomal positions. The results were expressed as a percentage (5632TH3 x PG2 +MTase versus 5632TH3 x PG2 control), with 100 % representing no difference; the lower the percentage, the greater the effect of MTase on mycoplasma chromosomal transfer.

### Correlation analyses between HGT and RM systems in *M. agalactiae*

Mobile elements present in *M. agalactiae* genomes were identified after annotation using the RAST server [31]. CDS annotated ‘mobile elements’ by RAST but also integrases, transposases, and prophage elements were counted, as well as genes identified as ICE by blast analyses against 5632 ICEA (e-value less than to 10^−3^). ICEs were considered when at least three of their essential components that are conserved across mycoplasma ICEs (CD5, CDS17, and CDS22) were present within a 30 kb DNA fragment. These were CDS5 and CDS17 encoding the conjugation factors TraE and TraG and CDS22 that encodes the DDE transposase [12]. Correlation analyses were performed with GraphPad Prism version 6.00 (GraphPad Software, San Diego, CA, USA).

## Results and discussion

### *M. agalactiae* genome-wide methylation landscape

A previous comparative genomic analysis conducted by our group suggested that *M. agalactiae* strains varied in their repertoire of RM systems [19], a situation that may impact their epigenomes differently. To gain insight into the epigenome of this species, DNA methylation profiles were first defined with two representative strains for which complete, circularised genomes were already available, namely 5632 and PG2. For this purpose, a combination of PacBio SMRT-seq and Illumina BS-seq, two complementary approaches, were used. SMRT-seq identified m6A and m4C modifications, while BS-seq detected both m4C and m5C. Intra-species diversity of the *M. agalactiae* pan-epigenome was further investigated by extending this approach to eight additional strains having different histories and collected over 30 years in France (Table 1).

Methylome data first indicated that the 5632 genome has twice as many methylated sites when compared to PG2, with a total of 26444 and 13268 sites detected, respectively. This observation correlated with 5632 having a higher number of predicted MTases than PG2 (Table 2) [19]. Examination of DNA methylations and their sequence context allowed the identification of 13 unique motifs when combining the results of the two strains (Table 3), six were assigned to the Type I RM system and seven to Type II and III. For both strains, the classical m6A adenine modification (dam methylation) was the most prevalent, as observed for other bacteria [29]. In contrast, cytosine modifications (dcm methylation) were detected in 5632 but not in PG2 and included the m4C modification identified by both SMRT- and BS-seq in the 5′-GC^m4^NGC-3′ motif, and the m5C modification that was only detected by BS-seq in the 5′-GGNC^m5^C-3′ (Table 3). Regardless of the methylation type, perfect palindromes were found to be methylated on both strands (dimethylated), while imperfect palindromes such as 3′-RCA^m6^C-5′and 3′-GA^m6^AG-5′ were hemimethylated (Table 4). A mix of both hemi- and dimethylated sites was observed for some Type II motifs, such as 5′-YRA^m6^TC-3′ and Type I 5′-HA^m6^YC(N)_5_KTAA-3′. Whether this situation reflects a difference in MTase affinity for their recognition sequence when more than one base is permitted at a particular position of the target site (degenerate recognition sequences) or differences in DNA methylation of both strands during DNA replication is not known.

**Table 3.** Methylated motifs detected in *M. agalactiae* 5632, 5632[hsd]_PG2_ (variant H1-2), and PG2 strains by SMRT and Bisulphite sequencing

					**5632**					**PG2**				
						**WT**		**[hsd]_PG2_**			**WT**		**5632**	**PG2**
	**Consensus**	**Detected motif**	**Modif**	**Predicted motif**	**Assignated methylase**	**Fract**	**nDetect**	**Fract**	**5632**	**Assignated methylase**	**Fract**	**nDetect**	**nGenome**	
**Type I**	AYC(N)_5_KTR / TRG(N)_5_MAY	HAYC(N)_5_KTAA	m6A	–	MAGa6290 MAGa6350	69%	761		1099				1099	
***hsd***		AYC(N)_5_GTR		–		64%	815		1266				1266	
		TTAM(N)_5_GRT		–		66%	831		1268				1268	
		YAC(N)_5_GRT		–		63%	793		1266				1266	
	AYC(N)_6_TRG / TRG(N)_6_CYA	AYC(N)_6_TRG	m6A	–				77%	1347	MAG5660 MAG5730	85%	980	1347	1063
		CYA(N)_6_GRT		–				68%	1554		74%	910	1554	1063
**Type II and III**	GANTC	GANTC	m6A	GANTC	MAGa7650	74%	4525	82%	6146	MAG6680	86%	4255	6146	4654
		GAAG	m6A	–	MAGa1580^#^	73%	8278	0	11 375				11 375	
	GATC	YRATC	m6A	GATC	MAGa2700	71%	5417	77%	7610 2590				7610	
		RGATC				67%	1732						2590	
	GGNCC*	GGNCC*	m5C	GGNCC	MAGa3950	95%	530	nd	530				558	
	GCNGC^†^	GCNGC^†^	m4C	GCNGC	MAGa4250	97%	2762	42%‡	2852				2852	
	RCAC	RCAC	m6A	–						MAG1530§	87%	7123		7758

All methylated motifs were detected by SMRT-sequencing except:

*Detected by BS sequencing.

†Detected by both SMRT and BS-seq, the fraction of BS-seq has been retained because due to intrinsic characteristics, SMRT sequencing is less confident for m4C detection.

‡This m4C fraction was only detected with SMRT sequencing, which explains why it is lower than those obtained with the same motifs detected by BS-seq (see above).

§Type III MTase

WT, Wild-Type; Fract, fraction nDetect: number of detected methylated motifs nGenome: number of motifs present in the genome

**Table 4.** Methylation status of detected motifs in 5632 and PG2 *M. agalactiae* strains

				5632		PG2	
	Consensus motif	**Detected motif**	Partner Motif String	**Dimeth (%**)	**Hemimeth (%**)	**Dimeth (%**)	**Hemimeth (%**)
**Type II**	**GANTC**	GANTC	GANTC	100	0	100	0
**&**	**GAAG**	GAAG	GAAG	0	100	na	na
**Type III**	GATC^*^	YRATC (77.5%)^#^		27.3	72.7	na	na
			YRATC	9.5	90.5		
			RGATC	100	0		
		RGATC (22.5%)^#^		98.2	1.8		
			YRATC	100	0		
			RGATC	94.5	5.5		
	**GCNGC**	GCNGC	GCNGC	100	0	na	na
	**GGNCC**	GGNCC	GGNCC	100	0	na	na
	**RCAC**	RCAC	none	na	na	0	100
**Type I**	**AYC(N)_5_KTR**	HAYC(N)_5_KTAA	TTAM(N)_5_GRT	88.2	11.8	na	na
		AYC(N)_5_GTR	YAC(N)_5_GRT	100	0		
	**AYC(N)_6_TRG**	AYC(N)_6_TRG	CYA(N)_6_GRT	na	na	100	0
		CYA(N)_6_GRT	AYC(N)_6_TRG				

*Proportion of detected motifs among ‘GATC’ type-sites.

†Total ‘GATC’ type motifs dimethylated = 33% and hemimethylated = 67%.

Hemimeth, hemimethylated; Dimeth, dimethylated; na, not applicable.

**Fig. 1.**
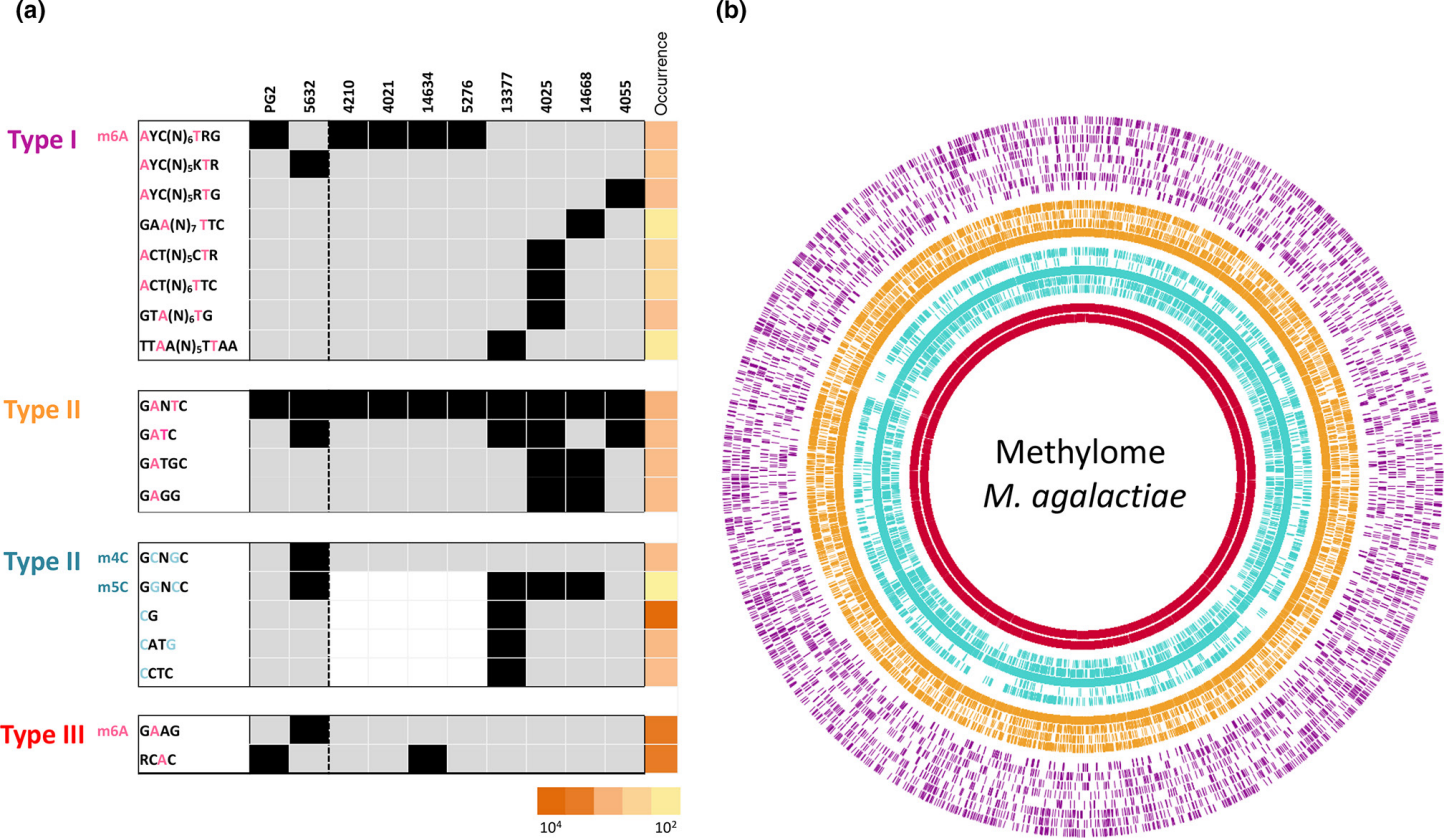
*
M. agalactiae* epigenome. (**a**) Heatmap representing the occurrence and distribution of methylated motifs detected by SMRT and BS-seq analyses across ten *M. agalactiae* strains. Motifs were grouped based on their assignment to Type I (purple), Type II (yellow for the Type II m6A methylated motifs and blue for the Type II m4C and m5C methylated motifs), and Type III (red) RM systems. The type of modification is also indicated: pink for adenosine methylation and blue for cytosine methylation. Strains not analysed by BS-seq are represented by a white box. The occurrence of methylated sites was evaluated *in silico* on the reference genome 5632 and is indicated by a column representing degrees of orange (the more intense the colour, the more the site is represented on the reference genome). (**b**) Circular plot representing the *in silico* position and occurrence of detected methylated motifs on the 5632 *M. agalactiae* reference genome. Methylated motifs were ordered as in the heatmap of panel A and grouped by Type and base modification. From outer to inner circle: Type I in purple, Type II m6a modification in orange, Type II m4C and m5C modification in blue, and Type III m6a modification in red.

Methylome data obtained with the panel of eight additional strains (Table 1) revealed the occurrence of both m6A and m5C methylated bases but not m4C, which remained specific to 5632 (Fig. 1). The occurrence of m4C is not uncommon in prokaryotes, with m4C and m5C accounting for 20 and 5 % of the modification types, respectively [29]. Whether m4C may confer a particular property to 5632 is discussed later. Overall, this extended analysis identified a total of 19 methylated motifs, of which 11 had not been previously detected in 5632 and PG2: eight were assignable to Type I RM and eleven to Type II and III RM systems (Fig. 1a). Of the detected motifs, 14 were associated with m6A modifications, with ten palindromic sequences that were methylated on both strands, while the other four imperfect palindromes (5′-GA^m6^TGC-3′, 5′-GA^m6^AG-3′, 5′-RCA^m6^C-3′, and 5′-GA^m6^GG-3′) were hemimethylated. Among those, two were Type III.

Based on their methylome profiles, the panel of ten *M. agalactiae* strains tested in this study could be divided into two main groups (Fig. 1). The first group is further referred as the PG2-like strains and included PG2, 4210, 4021, 14634, and 5276. Strains clustering in this group all had a limited number of methylated motifs and shared the same Type I recognition motif (5′-A^m6^YC(N)_6_TRG-3′) and the Type II 5′-GA^m6^NTC-3′ motif common to all strains. PG2 and 14634 strains also possessed the 5′-RCA^m6^C-3′ Type III methylated motif. The second group of strains (5632-like strains) included 5632, 13377, 4025, 14668, and 4055 and displayed more complex methylome profiles, with up to eight different types of methylated motifs for strain 13377. One particular feature of this group was the presence of Type II m5C MTase genes, as revealed by blast analyses against the dcm REBASE database [26], with that of 4055 being truncated (Table S1). In agreement with this observation, m5C methylations were detected in all 5632-like strains except 4055 and PG2-like strains (Fig. 1).

### *M. agalactiae* encodes sophisticated, variable, Type I RM systems

**Fig. 2.**
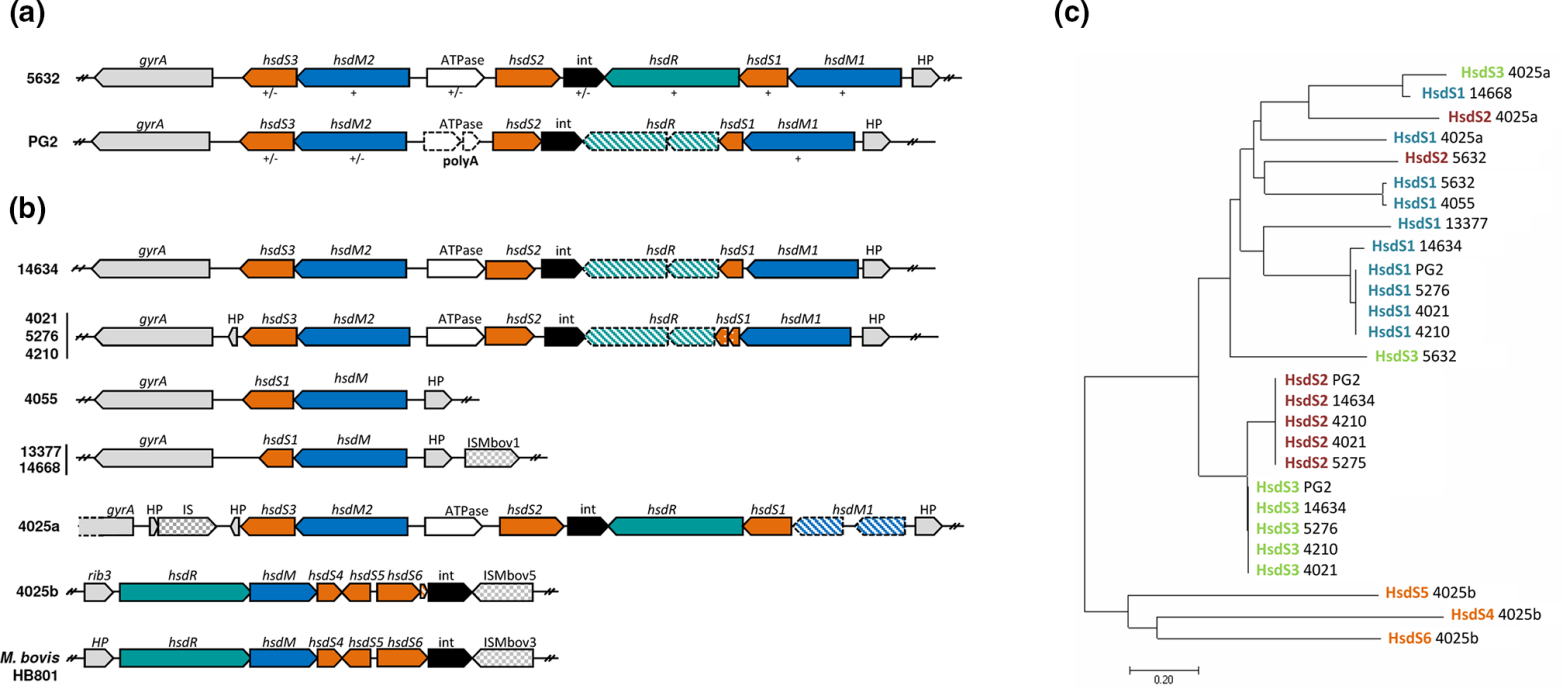
*M. agalactiae hsd* loci encode a Type I RM system. Schematic representing gene organisation of the *hsd* loci in (**a**) the two *M. agalactiae* reference strains 5632 and PG2 and (**b**) eight *M. agalactiae* field strains. Genes are indicated by an open arrow coloured based on the predicted function of their products (blue for methyltransferase, green for endonuclease, orange for DNA recognition subunit, black for integrase, and grey for other predicted gene). Pseudogenes are indicated by dotted lines and hatched genes correspond to truncated or size reduced genes. IS related transposase are indicated by grey squared arrows. (**c**) Evolutionary relationships of HsdS subunits. The evolutionary history was inferred using the neighbour-joining method based on a muscle alignment and the optimal tree is shown (sum of branch length=6.55524456). The tree is drawn to scale, with branch lengths in the same units as those of the evolutionary distances used to infer the phylogenetic tree. The evolutionary distances were computed using the Poisson correction method and are in the units of the number of amino acid substitutions per site. The analysis involved 27 amino acid sequences. All positions containing gaps and missing data were eliminated. There were a total of 36 positions in the final dataset. Evolutionary analyses were conducted in mega7 [67].

Type I RM systems are complex: in addition to restriction (R) and modification (M) subunits, Type I systems feature an additional specificity (S) subunit and work as multimeric complexes that recognise asymmetric sites. Typical Type I methylated motifs were detected in both 5632 and PG2 (Table 3) that consisted of a specific bipartite sequence separated by a 5N- and 6N-spacer, respectively. More specifically, in 5632, SMRT analysis identified four different, main motifs (Table 3) corresponding to six detected Type I methylated sequences (Table S3) and to the consensus sequence 5′–AYC(N)_5_KTR-3′ / 3′-TRG(N)_5_MAY-5′ with the target recognition domain (TDR) tandem TRD1=AYC/GRT (Y=C or T and R=A or G) and TRD2=KTR/YAM (K=G or T and M=C or A). Similarly, this analysis detected two different Type I motifs in PG2 corresponding to four methylated sequences and the consensus sequence 5′-AYC(N)_6_TRG-3′/3′-TRG(N)_6_AYC-5′, with TRD1=AYC/GRT and TRD2=TRG/CYA (Table 3 and S3). Type I specific methylated motifs were further detected in all *M. agalactiae* strains examined in this study (Table 3, Fig. 2). More specifically, strains of the PG2-like group, namely 4210, 4021, 14634, and 5276, all shared the Type I methylated motif 5′-AYC(N)_6_TRG-3′ found in PG2, while a greater diversity of sequences was observed for this motif in 5632-like strains. Notably, a single, specific Type I methylated motif occurred in all strains, except 4025, which had up to three different motifs (Fig. 1).

Several Type I systems have been described in mycoplasmas at the gene level and were designated as host-specific determinant (hsd) systems based on the seminal work performed in *M. pulmonis* [37]. In *M. agalactiae *, *hsd* loci were previously identified by blast analyses in strains 5632 and PG2 [19]. As depicted in Fig. 2(a), their overall organisation and synteny are similar, and each contained genes encoding the restriction enzyme *hsdR* (*hsdR* is truncated in PG2, see below) and several copies of the DNA modification (*hsdM*) and specificity (*hsdS*) components. blast analyses showed that amino acid sequences of HsdM were highly conserved, while those corresponding to HsdS differed within and between the two strains (Fig. 2a and S2). This raised the possibility that HsdS variability might be responsible for the Type I DNA diversity observed in 5632 and PG2. Type I sequences may be recognised by different HsdS subunits whose abundance, turnover, or affinity for the sequence may vary. In agreement with this hypothesis, the relative proportions of the detected TRD diverged from their theoretical abundance estimated *in silico* (Table S3), ranging from 7–31% and 16–44 % for 5632 and PG2 respectively (Table S3), while the proportion of different Type I detected motifs was comparable within each strain (63–69 % in 5632 and 68–77 % in PG2; Table 3). In *M. pulmonis *, phase variation through TRD ‘shuffling’ by recombination of the different *hsdS* genes has been demonstrated [37]. In this species, the Hsd system is composed of two distinct loci containing one *hsdR*, one *hsdM,* and two *hsdS*. DNA inversions involving *hsdS* genes can modify the DNA specificity of the resulting enzymes, generating up to 16 different DNA binding motifs [37]. In *M. agalactiae,* the organisation of the *hsd* locus resembles that of *M. pulmonis* with two *hsdM*, one *hsdR,* and three *hsdS* genes that occur as a single copy (Fig. 2). Whether *M. agalactiae hsdS* genes also undergo recombination to generate *hsdS* variation is not known, but such an event may explain the variability of the Type I profile detected in both 5632 and PG2 strains. Interestingly, a potential recombinase (‘int’ in Fig. 2) is encoded by the *M. agalactiae hsd* cluster, whose putative contribution toward DNA rearrangement within the locus remains to be addressed.

blast analyses further retrieved an *hsd* system in all *M. agalactiae* strains tested in this study (Fig. 2b). While their overall composition and organisation were similar, variations across strains were observed. Most strains contained at least three *hsdS* copies, being *hsd*S2 and *hsdS*3 highly conserved among PG2-like strains (Fig. 2 and S2). These strains displayed the same Type I motif, but as it was degenerate, more than one base was permitted at a particular position of the target sequences (Fig. 1a). This raised the question whether each *hsdS* subunit recognised a single sequence or whether there is a certain permissiveness of the subunits towards different sequences. A single *hsdS* gene copy was present in 13377 and 14668, whose respective Type I motifs corresponded to a single, distinct Type I sequence (Fig. 2). However, strain 4055, which also had a single *hsdS* copy, displayed a degenerate Type I sequence motif, but its mean score was among the lowest and the closest to the cut-off value, suggesting that this data may rather reflect a technical bias. At the other end of the spectrum, 4025 was equipped with six *hsdS* subunits distributed in two *hsd* loci: *hsd*4025a and *hsd*4025b (Fig. 2b). Interestingly, the number of detected Type I methylation sites was the highest in this strain with 1900 sites against 1600 in 5632 and 1050 in PG2, two strains having only each three *hsdS* coding sequences. SMRT analysis revealed that 4025 presented three Type I methylated motifs: TRD1=ACT/RTC, TRD2=ACT/CTT, and TRD3=GTA/GT, with either five or six nonspecific nucleotides as spacers (Fig. 1). Overall, these data suggested that strains equipped with a single HsdS/HsdM module tend to have a single, specific Type I methylated site and that the diversity of Type I methylated motifs correlates with the number of *hsdS* within one strain.

To further address the impact of domain specificity on *M. agalactiae* methylation, SMRT-seq was performed with a 5632 variant, namely H1-2, in which the complete *hsd* locus was replaced by that of PG2 (see Methods) [14]. Methylome analysis of the 5632 H1-2 variant revealed the occurrence of the PG2 Type I methylation profile, with TRD1=AYC/GRT, TRD2=TRG/CYA, and a 5N-spacer (see 5632 [hsd]_PG2_ in Table 3). Thus, replacement of the 5632 *hsd* locus by that of PG2 resulted in changing the Type I methylome profile of 5632 and supported that the *hsd* locus is responsible for Type I methylation, with variations of HsdS profiles being involved in modulating Type I targeted sequences.

The combination of multiple *hsdS* alleles with a potential recombinase gene in one locus provides a unique setting for *hsdS* sequence shuffling by DNA recombination, a phenomenon that, in turn, may drive recognition motif variations and dynamics in *M. agalactiae* species. In other mycoplasmas, Atack *et al.* (2020) [38] demonstrated that variation in the length of simple sequence repeats, such as AG[n], located in the HsdS coding sequence, was capable of mediating phase variation, thus leading to differential methyltransferase expression or specificity. Such repeats were not found in *M. agalactiae hsdS* genes, leaving gene ‘shuffling’ the best hypothesis for Type I variations.

*M. agalactiae hsd* clusters were always located at the same chromosomal position, downstream of *gyrA,* with the situation of 4025, which had an additional locus, *hsd*4025b, located elsewhere, downstream of the ribulose phosphate 3-epimerase *rib3* gene (Fig. 2b). The organisation of this locus is similar to the *hsd* locus of *M. bovis* strain HB0801, with 93 % identity, while the *hsd*4025a was closer to its counterpart in 5632. This suggested that *hsd*4025b was acquired from *M. bovis* by HGT, which was additionally supported by the occurrence of an *M. bovis* IS element, IS*Mbov5*, at one end of the *hsd*4025b locus (Fig. 2b).

Most *M. agalactiae* strains contained two *hsdM* genes, except for 13377, 14668, and 4055, which had only one (Fig. 2b and S2b). The extreme simplicity of the 13377 and 14668 *hsd* loci, which were only composed of an *hsdM*/*hsdS* module, the high similarity of their MTases with 5632HsdM1 (99.6 % nt identity for 14668, 99.5 % nt identity for 13377; Fig. S2b), and the occurrence of an IS-related transposase (IS*Mbov1*) near these loci suggested that these regions were subjected to evolutionary erosion (Fig. 2b).

Finally, *hsdR* was disrupted in PG2, as well as in the 4021, 4210, 5276, and 14634 genomes (Fig. 2b). This was due to the insertion of two nucleotides in a poly(A) region located in the middle of the gene that resulted in a premature stop codon ([19] and this study) and a severely truncated product. This implies that *hsdR* is not required for Type I methylation to occur in *M. agalactiae *. Furthermore, a complex of HsdS and HsdM was shown to be sufficient in *E. coli* for the preferential methylation of the target sequence at adenine residues, while the three Hsd subunits, M, S, and R, were required for DNA cleavage [39]. Truncation of *hsdR* also suggested that, in these strains, the Hsd system might be only capable of protecting DNA without restricting unprotected DNA. In mycoplasmas, polymeric tracts, such as poly(A) or poly(GA), are often prone to frequent insertion-deletion, a mechanism that could promote ON/OFF phase variation in the expression of the HsdR, as observed for the HsdS subunits in certain *Mycoplasma* spp. [38].

### Intra- and inter-strain diversity of the methylome produced by Type II RM systems in *M. agalactiae*

**Fig. 3.**
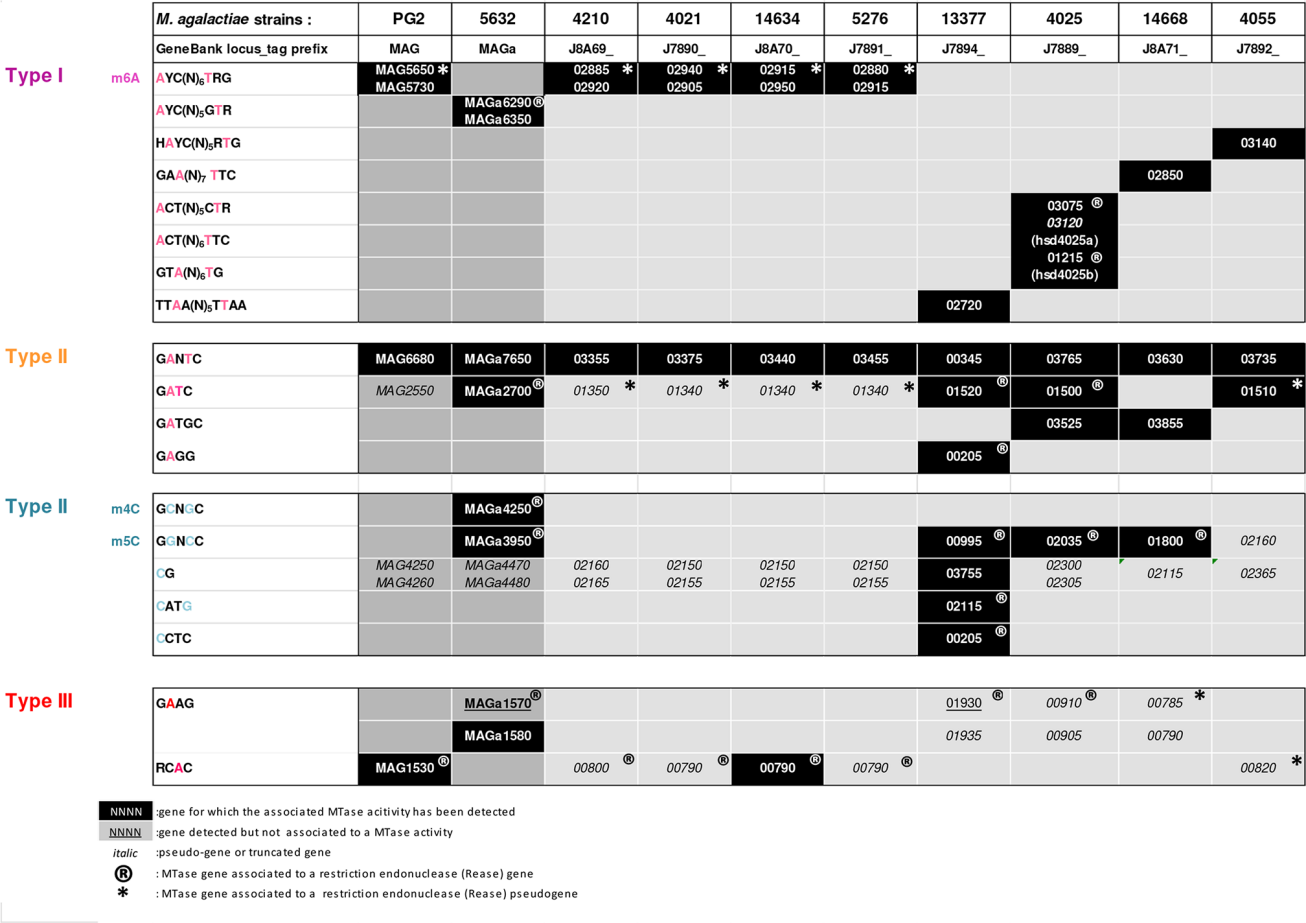
Methyltransferases assignation in *M. agalactiae* genomes. Methylated motifs detected by SMRT and bisulphite sequencing in ten strains of *M. agalactiae* and their associated methyl transferases. Motifs were grouped according to their assignment to Type I (purple), Type II (yellow for m6A and blue for m4C or m5C) and Type III systems (red). MTase gene name corresponds to Genbank accession number generated after automatic annotation of the gene using the Prokaryotic Genome Annotation Pipeline (PGAP) from NCBI. The Gene_tag prefix is mentioned in the second line. MTase genes that are associated with a cognate restriction endonuclease gene or pseudogene are indicated by a ‘R’ or by an asterisk, respectively. Black boxes correspond to genes for which the associated MTase activity has been detected. Underlined genes are those which have been detected genomes but for which no MTase activity has been detected. Pseudogenes or truncated MTase genes are shown in italics.

Type II methylated motifs are usually short palindromes [20] and were found in *M. agalactiae* with their occurrence, distribution, and sequences varying among strains, except for 5′-GA^m6^NTC-3′ which was detected in all strains. Based on REBASE prediction, this common motif can be associated with a Type II orphan MTase (Table 2) encoded by MAG6680 and MAGa7650 in PG2 and 5632, respectively, and by homologs in other strains (Fig. 3). In *Caulobacter crescentus* and other bacteria [22, 40], the GANTC motif is also recognised by an orphan MTase that methylates the adenine. This MTase belongs to the cell cycle-regulated DNA MTase family (CcrM) and plays an essential role in regulating the cell cycle. Whether the MTase encoded by MAG6680 and its homologs have a similar function in *M. agalactiae* remains to be demonstrated.

Two other degenerate Type II motifs, 5′-YRA^m6^TC-3′ and 5′-RGA^m6^TC-3′, were detected in 5632 but not in PG2. They were both assigned to a single MTase, MAGa2700, for which the predicted motif was 5′-GA^m6^TC-3′ (Table 3). Such an off-target methylation phenomenon has already been observed in *E. coli* for M.EcoKDam, a dam MTase involved in both chromosomal replication initiation and maintenance of genomic integrity [41]. Close examination of the 5632 methylome further revealed that 77.45 % of the GATC methylation corresponded to methylation of 5′-YRA^m6^TC-3′ (Y=C or T; R=A or G) and 22.55 % to the 5′-RGA^m6^TC-3′ motif, with 72.7 % of 5′-YRA^m6^TC-3′ being hemimethylated (Table 4). In all observed cases, this partial methylation occurred on one strand, usually the reverse strand, when the methylated site recognised was 5′-AA^m6^TC-3′ instead of 5′-GA^m6^TC-3′. The 5′-GA^m6^TC-3′ methylated motif was also detected in three other strains, namely 13377, 4055, and 4025, in which gene counterparts to MAGa2700 were identified (Fig. 3). In the remaining strains, homologs to MAGa2700 were either detected as pseudogenes or totally missing, as in 14668 (Fig. 3). In 5632, the MAGa2700 MTase is suspected to have a certain level of permissiveness regarding the recognition motif (see above), whereas in other strains the 5′-GA^m6^TC-3′ motif was the only detected sequence. Amino acid (aa) sequence alignment of products encoded by MAGa2700, and its homologs in strains 13377, 4025, and 4055, revealed two aa modifications out of 280 aa, in positions 22 and 97 of the active domain, which may explain the off-target methylation observed in 5632. Notably, 5′-GA^m6^NTC-3′ and 5′-GA^m6^TC-3′ were the most prevalent methylated motifs found among the PacBio SMRT sequenced *Mycoplasma sp*. of the REBASE Genomes database. This supports that these epigenetic modifications play an important biological role in mycoplasmas.

A third Type II m6 methylated site, the 5′-GA^m6^TGC-3′ motif, was detected in two strains, 4025 and 14668, and blast against the REBASE database retrieved a cognate MTase gene only in the genome of these two strains (see Fig. 3). These MTase coding sequences displayed 96 % identity with a putative Type II *N*6-adenine DNA methyltransferase of *M. bovis* PG45 (MBOVPG45_0722), which recognised the 5′-GCA^m6^TC-3′ motif, the reverse complement of GATGC, and corresponded to the *Sfa*NI prototype of *Streptococcus faecalis* (renamed *Enterococcus faecalis *) (REBASE database [26]). However, in 4025 and 14668, no cognate REase gene could be identified. Finally, the 5′-GA_m6_GG-3′ motif was only detected in the 13377 strain; the assignation of its corresponding MTase is discussed later.

The occurrence of Type II motifs with methylated cytosine, m4C or m5C, was also found in *M. agalactiae* but, as mentioned above, only in 5632-like strains. While all Type II methylated motifs detected in 5632 were at least present in one other strain of this group, the 5′-GC^m4^NCG-3′ was an exception, and m4C modification was only found in this strain. Using REBASE, this motif was associated with the MAGa4250 MTase in 5632, which had no homolog in the other strains. Four other Type II motifs with methylated cytosine were detected, all with m5C. Of these, the 5′-GGNC^m5^C-3′ motif occurred at least in four strains, namely 5632, 13377, 4025, and 14668, but was not detected in 4055. This motif was associated with the MAGa3950 MTase of 5632, homologs of which were encoded in 13377, 4025, and 14668 but was truncated in 4055 (Fig. 3). The three other cytosine-methylated sites, 5′-C^m5^G-3′, 5′-C^m5^ATG-3, and 5′-C^m5^CTC-3′, were all detected in strain 13377 but not in other strains of our panel (Figs 1 and 3). The case of the 5′-C^m5^G-3′ motif is addressed below, in the section dedicated to CpG methyltransferase. blast analyses using the REBASE database retrieved a cognate MTase gene for the other two motifs (Table 3 and Fig. 3). Interestingly, BS-seq data showed that the 5′-C^m5^CTC-3′ was methylated on the cytosine, while its reverse complement, 5′-GA^m6^GG-3′, was shown by SMRT analysis to be methylated on the adenine and was further classified as a Type II motif (see above). The dcm MTase responsible for 5′-C^m5^CTC-3′ methylation in 13377 is J7894_00205 (Fig. 3) and had 92 % identity with *M. bovis* M.MboH1ORF688P, a Type II cytosine-5 DNA methyltransferase, predicted to recognise the CCTC motif. Interestingly, the non-mycoplasma MTase that had the most significant homology with 13377 MTase J7894_00205 (63 % identity) was *Carnobacterium alterfunditum* M.Cal5972II, annotated as a m5C MTase. Yet, PacBio data suggested that this MTase is responsible for both the GA^m6^GG and T^m6^CCTCY methylations. In their REBASE comments, the authors suggested that methylation of this second motif indeed involved m5C on the complementary strand but that this modified base was not being called correctly (see REBASE PacBio data for M.Cal5972II). By analogy, our data strongly suggest that a single MTase, MTase J7894_00205, is responsible for the methylation of the two asymmetric motifs 5′-C^m5^CTC-3′ and 5′-G^m6^AGG-3 (Fig. 3). No dam MTase that could account for the m6A methylation of GAGG was detected in the 13377 genome, while the same motif was shown to be methylated differently. Such a situation is not unusual, and several studies have already shown the capacity of a given MTase to change its target base specificity, such as *EcoR*V in *E. coli* or *Pvu*II in *Proteus vulgaris* [42, 43]. For all Type II motifs with methylated cytosines described above, both cognate MTases and REases were identified (Fig. 3), suggesting that all Type II motifs except the CG motif (see below) were associated with an RM system.

Finally, three Type II MTase genes were initially predicted *in silico* for which no corresponding methylated motif was identified, namely CDSH of 5632 and MAG3310 and MAG4030 of PG2 (Table 2). However, none of their corresponding products were detected by proteomic analysis [19], indicating that these genes were most likely not expressed under our laboratory conditions or at undetectable levels. While MAG3310 and MAG4030 are encoded by the chromosome, CDHS is a putative Type II MTase carried by an integrative conjugative element, ICEA, occurring in three copies in the 5632 genome but absent from the PG2 strain. Previous data suggested a complex interaction among identical ICEA copies integrated in the same genome, including downregulation of some components [12]. The contribution of the putative CDSH MTase to the *M. agalactiae* methylome was then addressed in a PG2 derivative, in which a single ICEA copy was introduced by HGT, namely the PG2-ICEA+ variant (Table S4). Comparison of SMRT data obtained with PG2-ICEA+ and WT PG2 showed no difference, confirming that the CDSH MTase was not active under laboratory conditions regardless of the number of ICEA copies encoded by one genome. However, mating experiment data suggested that CDSH was expressed in PG2 when introduced behind a constitutive promoter (see below).

### *M. agalactiae* Type III methylome profiles and their possible association with phase variable RM systems

**Fig. 4.**
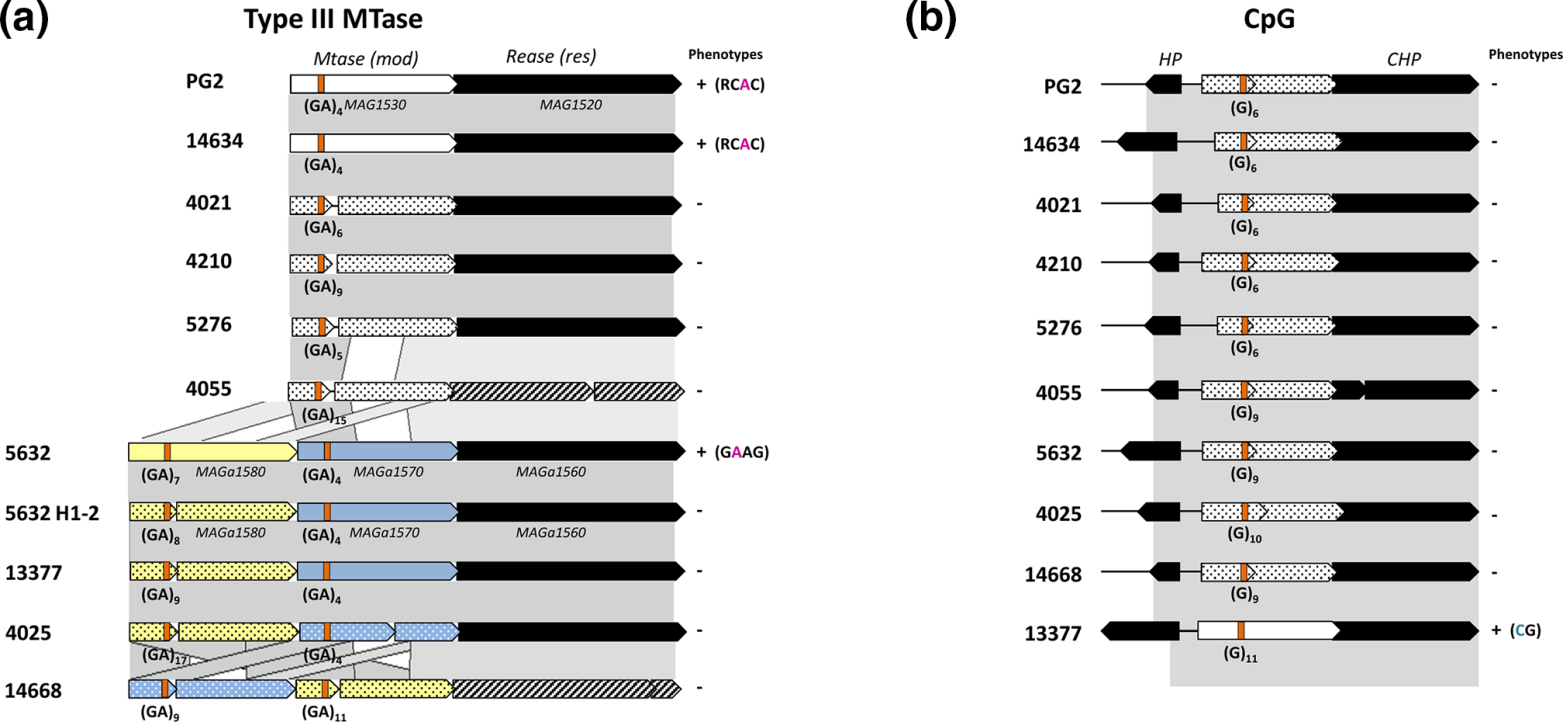
Type III RM loci and CpG methyltransferase in *M. agalactiae.* (**a**) Schematic representing the gene organisation and comparison between Type III RM systems identified in the ten sequenced *M. agalactiae* strains and in the 5632 H1-2 variant. Genes encoding Mod methyltransferase (MTase) and restriction endonuclease Res (REase) are indicated by white and black open arrows. Genes corresponding to truncated MTases are represented by open arrows filled with dots, and those corresponding to truncated REases are hatched. Poly(GA) tracts ([GA]) present in gene sequences are shown in orange, their most representative length was validated by Sanger sequencing (see Methods and Figures) and is indicated below the orange box. Detection of the associated methylated motifs, the ‘phenotype,’ is specified on the right by a ‘+.’ (**b**) Schematic representing the CpG MTase gene across the ten *M. agalactiae* strains. CpG MTase genes are indicated by white arrows filled with dots when the gene is truncated. Black open arrows represent unrelated flanking genes. The poly(G) ([G]) tracts present in gene sequences are shown in orange, and their length was validated by Sanger sequencing (see Methods) and is indicated below the orange box. Detection of the associated CG methylated motifs, the ‘phenotype,’ is specified on the right by a ‘+.’HP: Hypothetical protein, CHP : Conserved hypothetical protein.

Two different Type III methylated motifs were detected among the set of *M. agalactiae* genomes: 5′-GA^m6^AG-3′ and 5′-RCA^m6^C-3′. Both had a typical Type III motif, as they corresponded to dam methylation of short non-palindromic sequences. The 5′-RCA^m6^C-3′ methylated motifs was only detected in the PG2 and 14634 strains. These two strains harbour a predicted Type III MTase, designated as MAG1530 in PG2, and J8A70_00790 in 14634 (Fig. 3). Homologs were also detected in the other four PG2-like strains but occurred as pseudogenes because of a frameshift mutation at the beginning of their coding sequences that generated a premature stop codon (Fig. 4a; see below). Altogether, these data were in support of MAG1530 and J8A70_00790 as the best candidates to encode 3′-RCA^m6^C-5′ cognate MTases.

MAG1530 and its homologs had strong similarities with two 5632 CDSs predicted as Type III MTases (Table 2), namely MAGa1570 and MAGa1580 (64–70% identity). As shown in Fig. 4(a), these were organised as a tandem in 5632 and 5632-like strains, sharing ca. 65 % identity. Interestingly, methylation of the GAAG motif could only be detected in 5632 in which the two tandem genes were intact. In all the other 5632-like strains and in the 5632 H1-2 variant (Table 3), GAAG was non-methylated and a frameshift mutation was detected at the beginning of MAGa1580 (Fig. 4a). Altogether, these data designated MAGa1580 as the GAAG cognate MTase, but no methylated motif could be assigned to the predicted MAGa1570 MTase. As shown in Fig. 4(a), these MTase genes, alone or in tandem, are likely to form an operon with the downstream REase gene, thus constituting a Type III RM system. Type III RM systems are widespread in bacterial genomes and particularly in pathogens, in which they modulate genome methylation profiles by switching ‘on-off’ the expression of their MTases, an intra-clonal mechanism known as phase variation [44]. Furthermore, Type III *N*6-adenine DNA-methyltransferase encoding genes, known as *mod* genes, were previously described in mycoplasmas by Atack *et al*. (2018) [45] and more specifically in the *M. agalactiae* 5632 strain. In REBASE, these have been designated as M1 and M2.Mag5632ORF1570P and correspond to MAGa1570 and MAGa1580 of 5632, respectively, based on NCBI annotation. The features shared by *mod* genes include: (i) conserved 5′ and 3′ domains, (ii) conserved DPPY and FXXGXG Type III methyltransferase motifs required for function [44], and (iii) a variable central region encoding the TRD, which is responsible for the recognition of sequences methylated by the Mod protein. Upstream of this sequence, Atack *et al.* (2018) [45] identified a tract of [GA] repeats in which the variation in number was responsible for phase variation events. Our study showed the occurrence of a similar tract of [GA] repeats in the repertoire of *M. agalactiae mod* genes, with the number of repeats varying within and among strains from four to 17 (Fig. 4a).

In the six PG2-like strains, the *mod/res* locus was composed of a single MTase gene (MAG1530), followed by its cognate REase (MAG1520). In this group, Type III 3′-RCAm6C-5′ methylated sites were only detected in PG2 and 14634, an observation that correlated with the size of their poly(GA) being equal to four (Figs. 1 and 4a). In the other strains, homologs to MAG1530 were truncated due to length polymorphisms of [GA] repeats (Fig. S3) that resulted in a premature stop codon. The *mod/res* locus was more complex for 5632-like strains in which the locus was composed of two tandem MTases genes followed by a REase gene (Fig. 4a). In these strains, the two MTases genes often occurred as pseudogenes and all MAGa1580 homologs sequenced in this study were expected to produce truncated products due to length polymorphisms of [GA] repeats that generated a frameshift mutation and a premature stop codon. A similar situation occurred for MAGa1570 homologs of 14668 strain. In 4025 strain this homolog is truncated due to a deletion in the gene located downstream of the repeats (Fig. 4a). These observations suggest that both MTase genes are subjected to phase variation in expression due to length polymorphisms of [GA] repeats (Fig. S3). However, in our experimental conditions fragment length analysis (FLA) showed an active variation phase only for MAGa1580 homologs in strains 4025 and 14668 (Table S5). While detection of the 5′-GA^m6^AG-3′ methylated motif in strain 5632 but not in the 5632 H1-2 variant points towards MAGa1580 as the cognate MTase, the overall gene organisation of this cluster suggested that the two MTases might be necessary for methylation to occur. Indeed, Type III MTases are usually composed of a dimer of Mod subunits, where one Mod subunit recognises DNA, while the other Mod subunit methylates the target adenine [46].

Overall, differences in *mod* length polymorphisms of [GA] repeats combined with the analysis of the *M. agalactiae* methylome confirmed that the variations observed in this specific region had direct repercussions on the methylation status of mycoplasma genomes. In *M. agalactiae *, as in several other bacterial pathogens [45, 47], phase variation modulates the expression of Type III systems.

### Detection of an active CpG methyltransferase in *M. agalactiae*

BS-seq data revealed the occurrence of about 20.10^3^ 5′-C^m5^G-3′ motifs in a single strain of the panel, namely in strain 13377. CpG methylation is common in eukaryotes where it plays an essential role in controlling gene expression. In contrast, prokaryotic CpG methyltransferases have been exclusively described in the class Mollicutes, which includes *Mycoplasma* spp. [48–50]. In the AT-rich Mollicutes genome, the ‘CG’ motif is underrepresented, as observed in all *M. agalactiae* sequenced genomes, with a ‘CG’ abundance ranging from 0.46 and 0.5 (calculation based on the observed/expected ratio described by Goto *et al.* (2000) [51]; Table S6).

Data collected here showed that strain 13377 possessed an active CpG MTase for which a coding sequence, namely J7894_03755 (Fig. 3), was identified by blast analysis against REBASE dcm databases (Table S1). This coding sequence displayed 40 % identity with the MSssI and MmpeI CpG MTases found in two other Mollicutes, *Spiroplasma* spp. strain MQ1 and *Mycoplasma penetrans *, respectively [48, 49]. *Spiroplasma* MSssI was the first prokaryotic MTase described that specifically and exclusively methylates the 5′-CG-3′ sequence [48]. In this Mollicute, as in *M. agalactiae *, these CpG MTases methylate both strands, while their eukaryote counterparts preferentially generate hemimethylated DNA [52]. This is the first description of a ruminant pathogenic mycoplasma strain harbouring an active CpG MTase.

Comparative genome analysis revealed that all *M. agalactiae* genomes contained CpG MTase homologs (Fig. 3). A common feature of these coding sequences is that they all displayed a poly(G) region having between six and 11 ‘G’ located in their middle (Fig. S3). As shown in Fig. 4(b), the length of this poly(G) correlates with many of these gene products being truncated due to a premature stop codon. Only one strain, namely 13377, possessed an entire and active CpG MTase gene. Frameshift insertions-deletions occurring in CpG MTase genes have been previously described for other *Mycoplasma* spp. Moreover, in *M. pulmonis* or *M. crocodyli* [49], these were shown to modulate the length of a poly(GA) region also located in the middle of the CpG MTase sequence, as observed above for the poly(G). These poly(GA) or poly(G) stretches are located just upstream of the conserved and active domain of the dcm MTase (Fig. 4b). FLA analyses revealed that the phase variation is active in all tested *M. agalactiae* CpG genes (Table S5), with a variation of −1 to −2 nt within the polyG when compared to profiles present at more than 50 % in the total population, except for 14628 where the variation ranged from −1 to +1 ‘G’ . These data supported that expression and functionality of the mycoplasma CpG MTase active domain are controlled by frameshift mutations occurring in these regions and are thus phase variable.

In *M. hyorhinis *, CpG MTases and a CG overlapping GATC MTases (5′-CGAT^m6^CG-3′) were shown to translocate into the nucleus of human cells and efficiently modify their cellular epigenetic profiles [50]. The presence of an active CpG MTase may play a significant role in the survival, pathogenesis, and host adaptation of *M. agalactiae *, which is also capable of invading host cells [53]. Whether frequent, spontaneous mutations occurring in their coding sequences are stochastic mechanisms modulating MTase activity within clonal populations is not known, but the conservation of this phenomenon across *Mycoplasma* spp. indicates an important role.

### Abundance and distribution of methylated sites along the *M. agalactiae* genome

To gain insight into the putative role of DNA methylation in *M. agalactiae* biology, the distribution of methylated motifs in the chromosomes of strains 5632 and PG2 was analysed and compared. Data showed that all *in silico* predicted motifs were indeed methylated under our laboratory conditions. Motifs having m6A modifications, 5′-GA^m6^AG-3′ and the 5′-RCA^m6^C-3′ were the most abundant in terms of the number of methylated sites across all tested *M. agalactiae* strains (Fig. 1A and B). They were found every 100 bp, on average (about 10^4^ predicted sites in the 5632 genome of 1 Mb). Remarkably, the 5′-GA^m6^NTC-3′ motif was the only one present in all investigated *M. agalactiae* strains, with 2.4×10^3^ sites per 1 Mb of the 5632 genome. As for cytosine methylation, the 5′-C^m5^G-3′ was found every 100 bp on average for a 1 Mb genome and was thus the most represented m5C motif. All m4C and m5C methylated sites were present in an occurrence of about 10^3^/1 Mb, except for 5′-GGNC^m5^C-3′ which was less abundant (278 predicted motifs in the 1 Mb 5632-genome; Fig. 1a).

The distribution of predicted methylated motifs was further analysed using DistAMo (Fig. S4) [54], which determines motif distribution among genomes using codon redundancy to evaluate their relative abundance. This analysis revealed a bias towards the *oriC* and *ter* regions in 5632 for the 5′-GA^m6^TC-3′ (z-score: 1988) and the 5′-GGNC^m5^C-3′ (z-score: 2004) methylation motifs, respectively, while no bias was observed for PG2 (Fig. S4). DistAMo analyses also pinpointed the genes involved in cell cycle regulation as being overmethylated in both strains 5632 and PG2. The *ssb* gene, coding for a single-stranded DNA binding protein, displayed a high number of GANTC methylated motifs and the *hit* gene, coding for a HIT-like protein, in which Type III methylated motifs 5′-GAA^m6^G-3′ and 5′-RCA^m6^C-3′ were significantly overrepresented in 5632 and PG2, respectively (Table S7). The function of the *hit* gene is unknown, but proteins containing HIT domains form a superfamily of nucleotide hydrolases and transferases. This observation, together with the distribution bias of some motifs, supports the hypothesis that *M. agalactiae* DNA methylation plays a role in cell cycle regulation, as demonstrated for *C. crescentus* and other bacteria [22, 40].

The number of methylated motifs was also calculated in 1 kb windows along the PG2 and 5632 chromosomes (Fig. S4). No particular region or gene was under-methylated except for loci encoding the three copies of ICEA in 5632. Indeed, ICEA-encoded regions displayed a mean of 12.99 methylated motifs per 1 kb (out of a total 82 kb represented by the ICEs on the 5632 genome) for a calculated mean of 15.96 /kb for the 5632 genome (ranging from 3 to 39). Whether this difference has a biological impact is not known.

### Role of *M. agalactiae* RM systems in HGT

In prokaryotes, RM systems are known to provide a defence against foreign incoming DNA. Mycoplasmas are notoriously difficult to manipulate genetically, and this may in part be attributed to RM systems that prevent the entry of unprotected DNA. For instance, the 5632 strain can be efficiently transformed by specific mycoplasma vectors carrying the selective gentamicin or tetracycline resistance marker (pMT85, pminiO/T) [35]. Replacing these with the puromycin resistance gene (*pac*) designed by Algire and Lartigue (2009) [55] was successful in generating transformants using PG2 but not 5632. A close examination of the *pac* gene sequence revealed the presence of an Hsd-5632 recognition motif (5′-A^m6^YC(N)5KTR-3′) at the beginning of the gene (position 15/600, GenBank accession no. GQ420675.1). Failure in transforming 5632 with this vector carrying this sequence might thus be explained by the recognition and digestion of this non-methylated site by the REase subunit of the 5632-*hsd* system. Indeed, we successfully obtained puromycin-resistant transformants with the 5632 H1-2 variant (see above, data not show). Unlike the WT 5632, the H1-2 variant contained the *hsd* system of PG2, which one targets a different Type I motif and is predicted to produce a truncated, non-functional REase. These data highlighted the role of the Type I *hsd* system in the maintenance of foreign DNA in *M. agalactiae* once acquired, depending on the source.

MCT is an unconventional distributive conjugative mechanism responsible of HGT in *M. agalactiae *. In previous experiments, strain 5632 was always identified as the recipient cell regardless of the mating partners. This apparent polarity was true even when conjugation was bypassed by PEG-induced cell fusion, suggesting that the asymmetry of the DNA transfer might be independent of the conjugative mechanism itself, and relied on cytoplasmic factors that allowed, or not, the survival and/or the incorporation of foreign DNA [10, 11, 13, 14, 56].

**Fig. 5.**
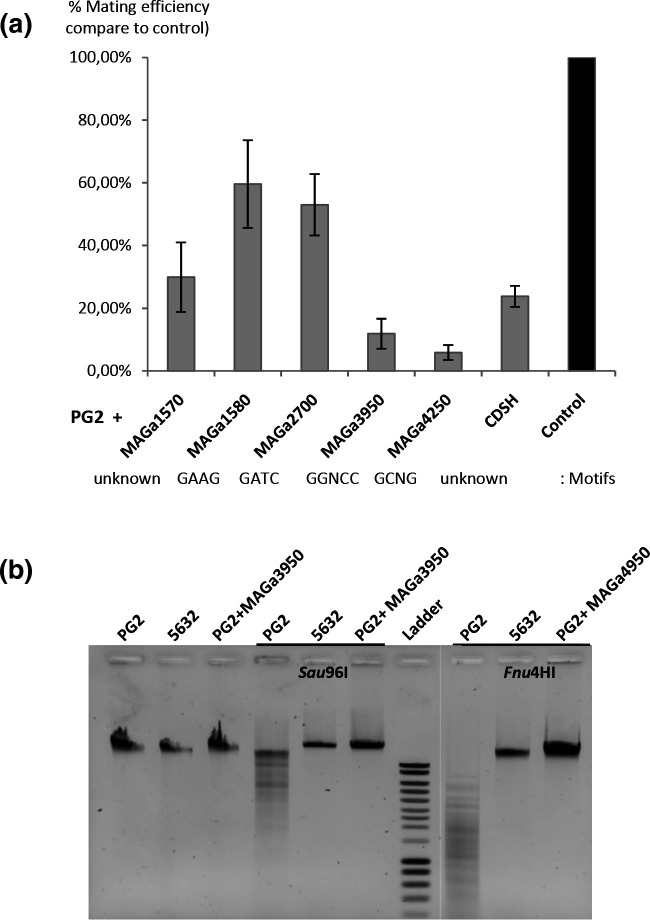
Contribution of *M. agalactiae* methyltransferases in mycoplasma chromosomal transfer. The PG2 strain was independently complemented by six MTase genes, absent in their active form in PG2 and originating from the 5632 strain, namely MAGa1570, MAGa1580, MAGa2700, MAGa3950, MAGa4250, and CDSH. The contribution of 5632 RM systems towards the acquisition of PG2 chromosomal DNA was addressed by comparing mating efficiencies obtained using 5632 and the individual complemented PG2 clones to that obtained with the non-complemented PG2 strain (represented by black bar plot, see Methods). Mating frequency of the control corresponded to 6±0.85 x 10^−8^ transconjugants/total c.f.u. For comparison purposes, results were expressed as a percentage of the ratio mating frequency assay/mating frequency control, with values close to 100 % representing no effect of the cloned MTase on DNA transfer on mycoplasma chromosomal transfer and those tending towards 0 % having the greatest effect. Bar plots represent at least three biological replicates, with error bars as the standard deviation between replicates. When possible, complemented MTase activities were controlled by restriction assays as illustrated in (**b**) by agarose gel electrophoresis. DNA extracted from 5632, PG2, and complemented PG2 strains were restricted by commercialised restriction enzymes *Sau*96I and *Fnu*4HI, which targeted the motif corresponding to the MAGa3950 and MAGa4250 MTases, respectively.

To test this hypothesis and evaluate the impact of DNA methylation on DNA transfer polarity, mating experiments were conducted using 5632 and PG2 complemented by either one of the six Type II/III MTase genes identified above as being specific of 5632. These were MAGa1570, MAGa1580, MAGa2700, MAGa3950, MAGa4250, and CDSH (see Tables 2 and 3). Complemented MTase activities were controlled by restriction assays using DNA extracted from complemented PG2 strains and commercialised restriction enzymes, when available, that targeted the motif corresponding to the tested MTases (*Sau*96I for MAGa3950, *Fnu*4HI for MAGa4250, and *Dpn*I/*Dpn*II for MAGa2700; see Methods; Figs 4 and S1). Restriction profiles confirmed that MAGa3950, MAGa4250, and MAGa2700 of 5632, once introduced into PG2, protect DNA from being restricted. These data demonstrated that MTases were expressed and active in complemented PG2 clones and further confirmed that the detected methylated motifs were correctly assigned to the MTases in 5632. Notably, no commercialised REase matched the motif recognised by MAGa1580. Complemented PG2 clones were individually mated with WT 5632, and their mating efficiencies were compared to those obtained in parallel with a pair composed of the parental strains, WT PG2 and WT 5632, as controls. The results were expressed as a percentage, where 100 % represented no difference and thus no effect on HGT of the MTase expressed by the complemented-PG2. As shown in Fig. 5, a decrease in mating frequency was observed with all complemented-PG2, regardless of the MTase genes used. The most important effects were observed when using PG2 complemented with MAGa3950, MAGa4250, MAGa1570, and CDSH, with a decrease of at least 70 % of the mating frequency compared to the control. Of these, the MAGa4250 MTase had the most significant impact, showing a 95 % decrease. Complementation of PG2 with CDSH under the control of a strong constitutive promoter (promP40), resulted in a reproducible, reducing effect towards the transfer of PG2 fragments into the 5632 genome. In strain 5632, CDSH is encoded by ICEA and was previously suspected to be downregulated in standard growth conditions [12]. Our current results suggested that (i) CDSH is expressed once placed behind a constitutive promotor, and (ii) CDSH may contribute in protecting DNA either from being transferred or incorporated into the recipient 5632 strain. Finally, the MAGa1580 and MAGa2700 gene products had a less significant impact, with only a 40 % decrease in HGT efficacy.

On one hand, part of our data supports the general idea of RM systems providing a protective barrier to HGTs by digesting non-methylated, foreign DNA. On the other hand, some RM systems might be key in MCT by fragmenting the unmethylated, chromosomal ‘donor’ DNA, thereby facilitating its incorporation into the methylated ‘recipient’ chromosome. Several studies have indeed pointed towards restricted DNA products as stimulating homologous and non-homologous recombination with the host genome [57]. In the case of this study, such a hypothesis could explain the apparent polarity repeatedly observed during chromosomal exchanges, with the 5632 strain always acting as the recipient cell when mated with a strain underequipped in RM systems such as the PG2 strain. Indeed, 5632 was shown to be the most methylated strain, with a total of 26444 methylated sites (Table 3). It is also one of the richest in terms of restriction endonucleases, with five REases associated to RM systems (see Fig. 3). Interestingly, 5632 was the only strain of our panel harbouring the 5′-GC^m4^NGC-3′ motif. Once artificially introduced in the PG2 strain, its cognate MTase, MAGa4250, provided the complemented strain with an epigenetic modification which was the most effective in preventing MCT (Fig. 5). This suggested that the RM system associated with this motif, and most specifically the REase, might be key in MCT and thus in genome dynamics.

### Correlation between RM systems and the *M. agalactiae* mobilome

The interplay between bacterial RM systems and MGE is complex. Often considered as a barrier to invading MGE, RM systems can also stabilise MGE in cells by preventing infections by other competing MGEs [18]. The association of MGEs and RM systems might result from an increased selection of RM systems facing foreign MGEs. As mentioned above, REases of these systems could also favour HGT by producing double-stranded DNA ends that are recombinogenic [18, 57, 58]. In this case, genomes enduring more HGT and harbouring more MGEs would have more RM systems.

**Fig. 6.**
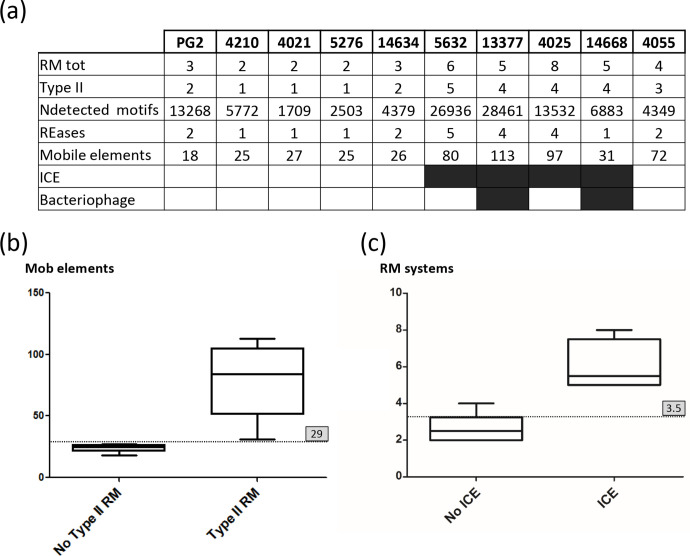
Correlation between horizontal gene transfer and restriction modifications systems in *M. agalactiae *. (**a**) Number of restriction modification systems (RMtot), Type II RM systems (Type II), methylated motifs (Ndetected motifs), restriction endonucleases (REase), and mobile elements detected in the ten strains of *M. agalatiae*. The number of mobile elements includes CDS annotated by RAST (30) as mobile elements, integrases, transposases and prophage elements and genes identified by Blast against 5632 ICEA (e value less than to 10^−3^) as belonging to ICE elements. Based on previous work (59), elements were counted as ICE when at least the following three main components were present and complete: CDS5 and CDS17 encoding the conjugation factors TraE and TraG, respectively, and CDS22 encoding the integrase. (**b**) Boxplot showing the strong correlation between the presence of Type II active RM systems (excluding the 5′-GANTC-3′ methylation found in all strains) and the number of mobile elements (Pearson’s r=0.78, *P*=0.008) in *M. agalactiae* genomes. (**c**) Boxplot showing the very strong correlation between the presence of ICE elements and the number of RM systems in *M. agalactiae* genomes (Pearson’s r=0.86, *P*=0.001). For both boxplots, the dotted line represents the median; their values are indicated in the grey box on the right.

To evaluate the co-occurrence between MGEs and RM systems in mycoplasmas, the presence of MGEs in the ten *M. agalactiae* genomes included in this study was investigated. For this purpose, for each strain, the repertoire of CDS related to mobile genetic elements (MGE), such as transposases commonly found in IS or integrase found in ICE and prophages, was analysed. MGE-associated CDSs were found in each genome, ranging from 18 to 113 per genome (Fig. 6a, Table S8). Additionally, the minimal backbone of functional ICEs, with CDS5 and CDS17 encoding for TraE and TraG conjugation factors and CDS22 encoding for the ICE DDE-transposase [59], were retrieved in four strains (5632, 4025, 13377, and 14668; Fig. 6a). Vestigial ICE forms were detected in nearly all strains, except 4055. Despite the paucity of prophage sequences in mycoplasmas, this study also retrieved a prophage in two strains (13377 and 14668). Thus far, *M. agalactiae* prophages have only been detected in strains isolated from ibex [60].

These data were used to evaluate the correlation between the presence of RM systems, the number of methylated sites detected (methylome), and the presence of MGE-related CDSs in the genomes of *M. agalactiae *. Our analysis revealed a strong correlation between active Type II RM systems and the presence of MGEs in *M. agalactiae* genomes (Pearson r=0.78, *P*=0.008). This correlation was also significant when all RM systems were considered (Pearson r=0.71, *P*=0.02; Fig. 6b) and was even stronger when our analysis was restricted to ICE elements, which are an important contributor to HGT in mycoplasmas (Pearson r=0.86, *P*=0.001; Fig. 6c) [10, 12]. Of note, the Type II GANTC methylation that was broadly distributed across all tested strains and was suspected to be involved in cell cycle regulation, was not considered in this analysis.

Our results agreed with previous genomic studies that indicated a positive association between HGT capacity (based on MGE abundance in genomes) and the presence of RM systems, especially in larger bacterial genomes [18]. This observation was made here with mycoplasmas, which are renowned for having some of the smallest bacterial genomes and is in opposition with the classical view in which RM systems contribute to bacterial immunity by protecting bacteria from exogenous DNA. To overcome this apparent contradiction, Oliveira *et al.* (2016) [18] proposed in one of their hypotheses that RM systems may favour the transfer of genetic material between cells by generating restriction breaks that stimulate recombination between homologous sequences. This scenario is consistent with the strong correlation observed between the abundance of REases and MGEs present in the *M. agalactiae* genomes (Pearson r=0.848, *P*=0.0019; Fig. 6b, c).

ICEs are intimately linked to HGT in mycoplasmas. They provide these bacteria with the capacity to conjugate, thus allowing them to exchange a large amount of chromosomal DNA via a conjugative, distributive mechanism designated as MCT. This process involves swapping large portions of the recipient mycoplasma genome by the donor counterparts via homologous and non-homologous recombination events [14]. Thus, these exchanges of DNA materials may be facilitated by the presence of endonucleases, which would provide restriction breaks that stimulate recombination and promote HGT between cells.

### Occurrence of *M. agalactiae* MTases outside the species

**Fig. 7.**
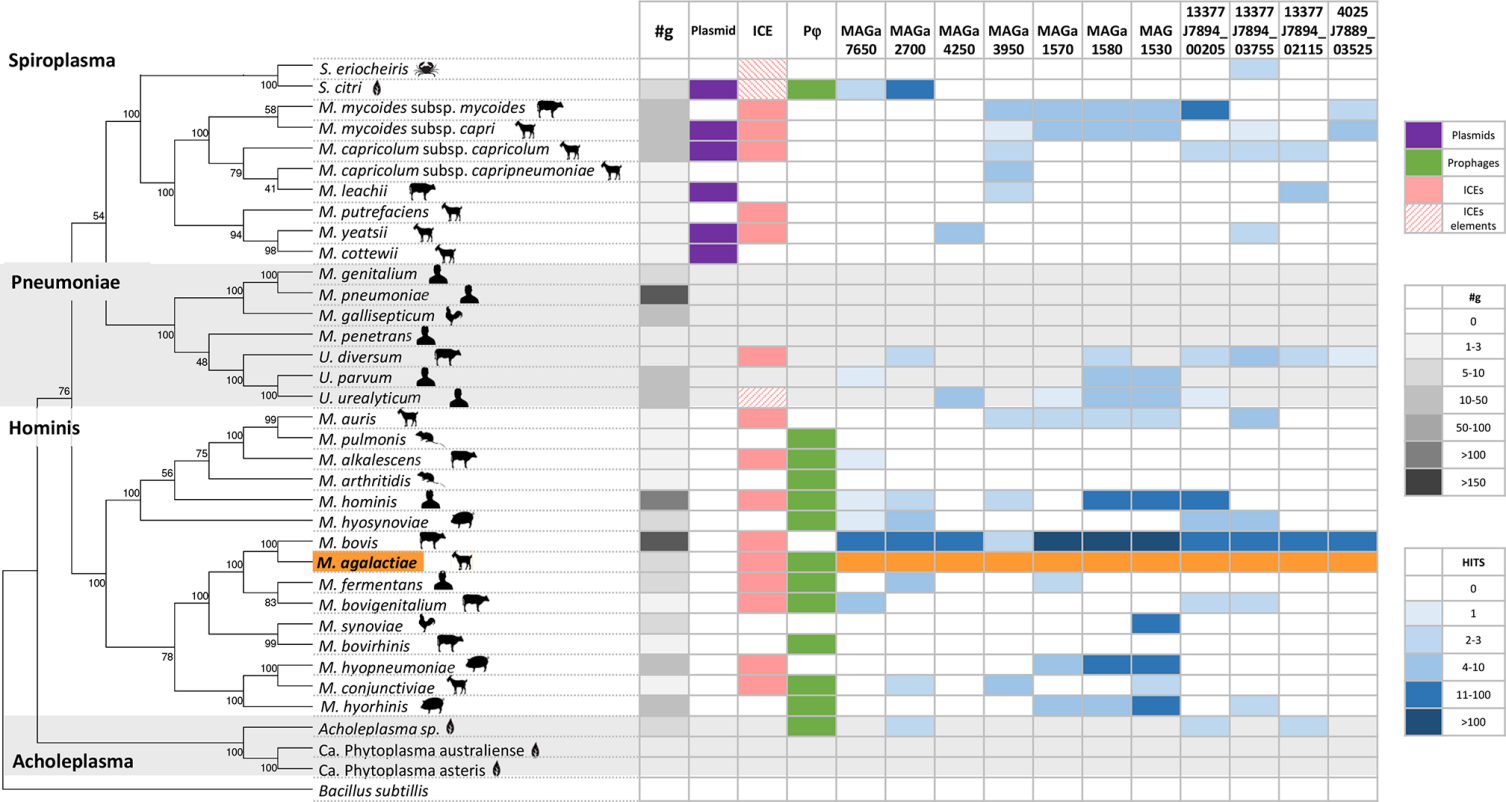
Distribution of active *M. agalactiae* methyltransferases among Mollicutes. Phylogenetic tree based on 16S ribosomal DNA sequences of 36 major representatives of Mollicutes aligned with clustalW (mega 7; 67). The evolutionary history was inferred using the neighbour-joining method. The evolutionary distances were computed using the Poisson correction method. There were a total of 36 positions in the final dataset with a bootstrap of 500 replicates (percentage indicated next to each branch). *Bacillus subtilis* was used as an extra group. The main four phylogenetic groups are shown in the left. Our study model *M. agalactiae* is highlighted in orange. For each mollicute species, their host is symbolised next to their name. The proportion of sequenced genomes available in current databases are indicated in the ‘#g’ column by a grey gradient (the higher the number of genomes, the darker the grey). The presence in each species of plasmids, prophages, full length ICEs, or ICE elements is represented by purple, green, pink, and hatched pink boxes, respectively. The occurrence and number of active *M. agalactiae* methyltransferases present in other Mollicutes are indicated by blue boxes. They were determined by BLASTP analyses (NCBI Taxonomy results) on *M. agalactiae* methyltransferase protein sequences against Mollicutes genomes available in current databases. The blue gradient represents the number of positive hits found after BLASTP analysis for each species (the higher the number of results, the darker the blue).

Whether MTases identified above were specific to *M. agalactiae* was addressed by BLASTP analyses against bacterial genomes available in current databases (see Methods). Furthermore, the five following MTase genes had no homolog outside of the Mollicutes class, which includes *Mycoplasma* spp., as well as *Ureaplasma *, *Spiroplasma *, and *Acholeplasma* spp.: the CpG methyltransferase, as previously described (see above), the Type III related MTases (MAGa1570, MAGa1580, and MAG1530), and the Type II MTase of 13377, J7894_02115 (Fig. 3, Table S9). Of the 11 Type II and III MTases identified in *M. agalactiae *, all were found in at least one other Mollicute, of a different species or genus and of a different phylogenetic group (Fig. 7, Table S9). There was no correlation between their distribution and the phylogenetic cluster, the genome size, or the host of the species; however, this must be considered with care since the number of strains with sequenced genomes varies among Mollicutes. As shown in Fig. 7, a remarkable observation is that homologs to these MTases were totally missing in eight *Mycoplasma* spp. For some, namely *M. cottewii *, *M. penetrans *, *M. pulmonis *, *M. arthritidis,* and *M. bovirhinis *, this may be due to the limited number of sequenced strains (*n*<3). However, this explanation is not valid for *M. pneumoniae* and *M. gallisepticum,* for which over 150 genomes are available for each and unlikely for *M. genitalium* with six strains sequenced. These three *Mycoplasma* spp. that have different hosts all clustered in the Pneumoniae phylogenetic group. Remarkably, in these species no ICE was detected, and the *M. gallisepticum* genome carries an active CRISPR/Cas system [61], a bacterial adaptive immune system that protects from invasion by foreign DNA [62].

Finally, six MTases displayed significant homology outside of the Mollicutes, with some having up to 70 % identity (coverage 100%) with CDS annotated as DNA methyltransferases in bacteria that are phylogenetically distant (Table S10). Altogether, our data indicate that *M. agalactiae* MTases identified in this study, and most likely their cognate REases when belonging to RM systems, frequently occurred outside of the species within the Mollicutes and outside of this particular class, most likely as a result of HGT.

## Conclusion

This study provides a comprehensive insight into the genome-wide methylome of several *M. agalactiae* strains at single-base strand resolution using a combination of PacBio SMRT- and BS-sequencing. When combined with whole-genome analysis, this approach identified 19 methylated motifs associated with three orphan MTases and eight RM systems.

A single motif, the Type II G^m6^ANTC motif, was detected across all *M. agalactiae* strains tested here. This motif was associated with an orphan MTase having some similarities with the cell cycle-regulated DNA MTase family (CcrM) and is likely to play a role in basic, biological mycoplasma function(s). Two other Type II motifs were associated with orphan MTases, namely the G^m6^ATGC, which was detected in only two strains and for which there was no hint regarding a potential function, and the C^m5^pG which was only detected in one strain. In prokaryotes, CpG methylated motifs are exclusively found in the class Mollicutes, which includes *Mycoplasma* spp. The role of this methylation in mycoplasmas is not known, but studies conducted with *M. hyorhinis *, a facultative, intracellular pathogen of swine, suggested that this bacterium is able to deliver CpG methyltransferase into eukaryotic cells and selectively methylate the host genome, thus impacting its epigenome [50]. Whether *M. agalactiae,* which is also described as facultatively intracellular, modulates its host epigenome remains to be elucidated. In *Mycoplasma* spp., CpG MTase is likely to be regulated at the population level, during clonal propagation, via high-frequency phase variation. Furthermore, a CpG MTase gene was found across all *M. agalactiae* strains that displayed a hot spot for spontaneous mutations typical of such a system. Orphan MTases were likely derived from RM systems by loss of the cognate REases at an early evolutionary stage history of the species [63]. Some, such as the CcrM-like or CpG MTases, may be domesticated by the host chromosome to serve essential functions, thus becoming part of the core-genome of the species; others might have been further disseminated via HGT [17] as part of the variable pan-genome.

Most methylated motifs identified in this study were associated with the Type I *hsd* system described in other *Mycoplasma* spp., notably in *M. pulmonis* [37]. In this species, the specificity of this system for a particular target sequence was shown to undergo phase variation following DNA shuffling within the *hsd* locus. The *hsd* genetic organisation and the diversity of the Type I m6A sequences identified in this study suggest that the same phenomenon is occurring in *M. agalactiae,* in addition to gene erosion and gene transfer. MTase expression of two other Type II and III RM systems identified here is undergoing phase variation based on frameshift mutations occurring in a homopolymeric tract, poly(G) or poly(GA). Such a genetic mechanism has been extensively described in mycoplasmas, in which it controls ‘on-off’ switching in the expression of a variety of products [64].

Several phase-variable DNA methyltransferases have been identified in major human pathogens and shown to control the expression of multiple genes via epigenetic mechanisms. These systems also called phasevarions, for phase-variable regulons, regulate genes involved in pathogenesis, host adaptation and antibiotic resistance [45, 65]. Some of the phase-variable systems identified in the current study are likely to play a similar role in *M. agalactiae* and would provide this pathogen with a means to compensate the lack of gene regulators found in more classical bacteria.

Based on their methylome profiles, the panel of *M. agalactiae* strains could be equally subdivided into two groups. One displayed only m6A methylated motifs together with a limited set of MTases and RM systems. The other group was more complex and diverse, including both methylated cytosine (m4C and m5C) and adenine (m6A) motifs, along with several RM systems and a CpG methyltransferase. The m4C is not a rare DNA modification in bacteria, but to our knowledge, this methylation has never been reported in mycoplasmas [28, 29]. Here, m4C was only detected in the GCNGC motif of strain 5632, and in agreement with this finding, this strain was the only one equipped with the cognate MTase gene, namely MAGa4250. *M. agalactiae* strain 5632 can uptake and integrate chromosomal DNA from other strains via a conjugative, distributive mechanism designated MCT, which remains to be fully understood. This phenomenon was rarely observed with other strains, and when it occurred, it was at low frequency and in strains that were genetically similar to 5632. One hypothesis was that 5632 had the capacity to incorporate the incoming chromosomal DNA only once digested. In support of this hypothesis, our data indicated that cloning MAGa4250 into PG2 almost abolished the incorporation of PG2 chromosomal DNA into 5632, most likely by protecting the PG2 chromosomal DNA from being digested by the MAGa4250 MTase cognate REase. A search across the mycoplasma database (data not shown) retrieved a MAGa4250 homolog only in a few strains of *M. bovis *, a close relative of *M. agalactiae *, and in two other ruminant *Mycoplasma* spp., *M. californicum* and *M. yeatsii *. Whether the rare occurrence of this MTase across Mollicutes is due to the low number of sequenced genomes for some species is not known. Thus far, the MAGa4250-RM system has been found in two distant phylogenetic groups in *Mycoplasma* spp. having the ruminant host as a common factor. This observation suggested that this system might disseminate via MCT across species sharing the same ecosystem, in addition to being a key player in this process. In *M. agalactiae *, RM systems may play a central role on MCT and on the polarity of DNA transfers in offering a delicate balance between the protective effect of MTases and the facilitation of DNA incorporation into the host chromosome by REases.

Overall, several superimposed genetic events may participate in generating a dynamic epigenome landscape within and among *M. agalactiae* strains. These occur at loci encoding orphan MTases or RM systems and include (i) DNA shuffling and frameshift mutations that affect the MTase and REases content of a clonal population during propagation and (ii) gene duplication, erosion, and transfer that modulate MTase and RM repertoires across the species. In turn, the versatility of these systems may contribute to regulating essential biological functions and virulence factors at both cell and population levels, and they may be key in mycoplasma genome evolution and host-adaptability by controlling gene flow among cells.

## Supplementary Data

Supplementary material 1

Supplementary material 2
