# Supplementary material for: Impacts of Mycoplasma agalactiae restriction-modification systems on pan-epigenome dynamics and genome plasticity
Source: Microb Genom. 2022 May 16;8(5):mgen000829. doi: 10.1099/mgen.0.000829 (PMC9465063; doi:10.1099/mgen.0.000829)
Supplement: Supplementary material 1 [file mgen-8-829-s001.pdf]

SUPPLEMENTARY DATA

Figure S1. Patterns obtained after agarose gel electrophoresis of restricted DNA extracted from PG2 strain complemented with methyltransferases originating from the 5632 strain

Figure S2. Evolutionary relationship of HsdS, HsdM, and HsdR of the ten *M. agalactiae* tested strains

Figure S3. Electropherograms regions corresponding to repeat tracts observed in genes encoding CpG and TypeIII Methyltransferases

Figure S4. Distribution of *M. agalactiae* methylated motifs along the 5632 and PG2 chromosomes

Table S1. BLAST analysis against dcm REBASE database

Table S2. Primers used for PCR and Sanger sequencing

Table S3. Percentage of methylated TRD couples corresponding to Type I RM systems detected by SMRT sequencing compared to *in silico* motif abundance in 5632 and PG2 strains

Table S4. Methylated motif detected in PG2 and PG2 ICEA+ variants

Table S5. Fragment length analysis (FLA) of *M. agalactiae* Type III and CpG methyltransferases

Table S6. CG abundance *of M. agalactiae* tested strains

Table S7. Over- and under methylated genes based on DistAMo analysis in *M. agalactiae* 5632 and PG2 stains

Table S8. Mobilome of the ten sequenced *M. agalactiae* strains

Table S9. BLASTP hits obtained for *M. agalactiae* active methyltransferase against the current Mollicutes genome database.

Table S10. BLASTP results for *M. agalactiae* active methyltransferases against bacteria other than the Mollicutes class;

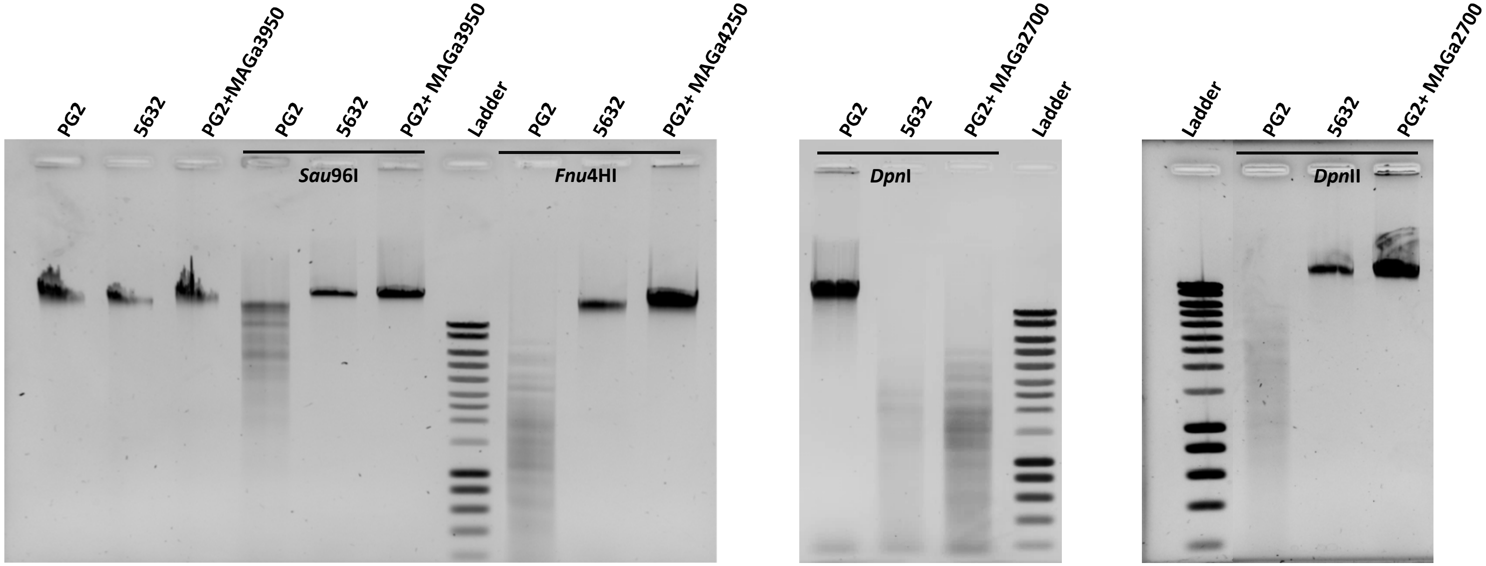

Figure S1. Agarose gel electrophoresis of DNA restrictions of PG2 strain complemented with methyltransferases originating from the 5632 strain. Complemented activities were controlled by restriction assays when commercial restriction enzymes were available as illustrated in the agarose gel electrophoresis presented here. The DNA extracted from 5632, PG2 WT and complemented PG2 strains were restricted by commercialized restriction enzymes *Sau96*I , *Fnu4H*I, *Dpn*I and *Dpn*II which targeted the motif corresponding to the MAGa3950 and MAGa4250 MTases and MAGa2700 (for both *Dpn*I and *Dpn*II), respectively. *Dpn*I and *Dpn*II recognize the same sequence but have different methylation sensitivities. *Dpn*I will only cleave fully adenomethylated dam sites and hemi-adenomethylated dam sites 60X more slowly. *Dpn*II cleave dam sites that lack adenomethylation and is blocked by complete dam methylation.

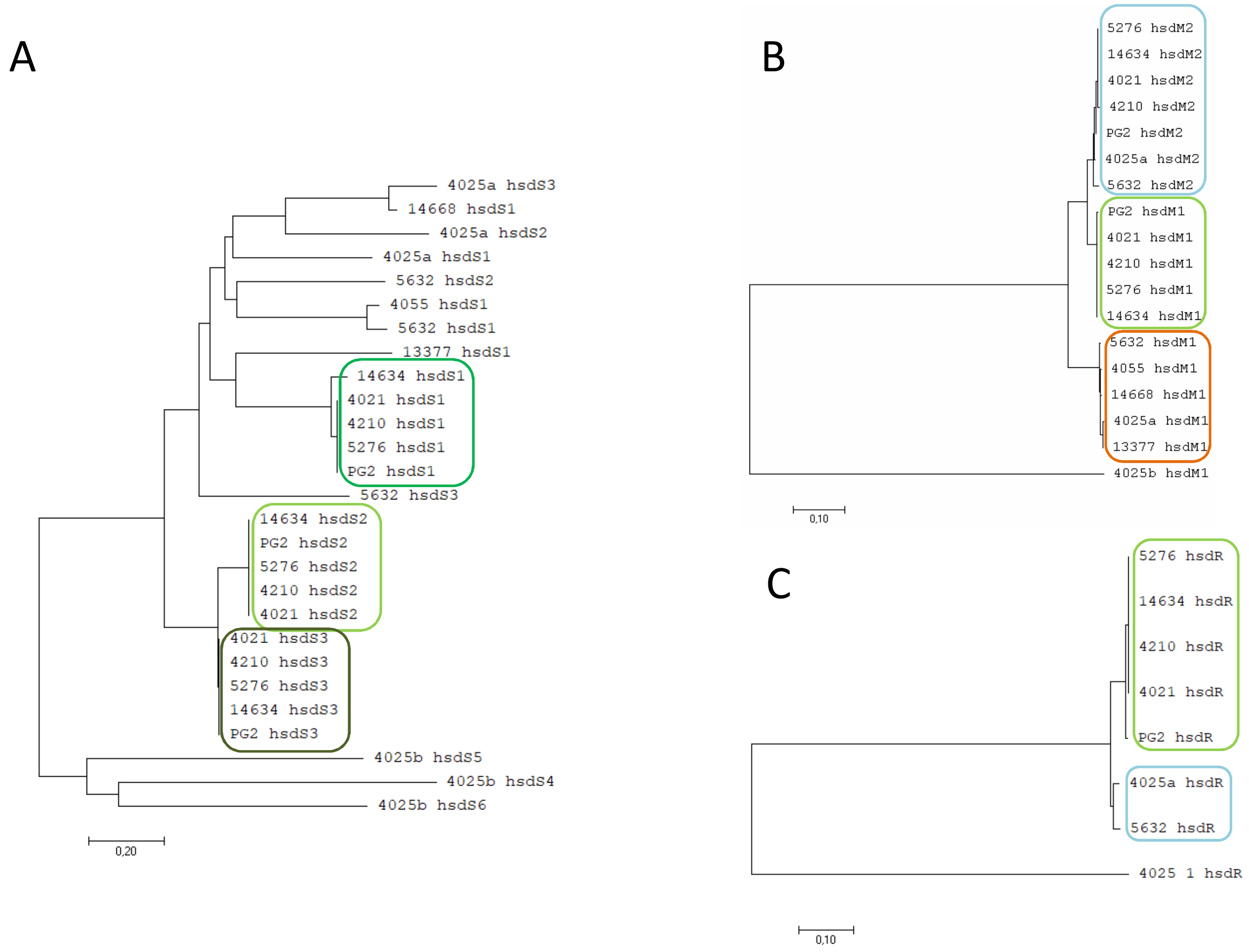

Figure S2 Evolutionary relationships of HsdS, HsdM, and HsdR of the ten *M. agalactiae* tested strains

Neighbor-Joining phylogenetic trees of the several *M. agalactiae* Hsd subunits. Proteins were aligned with MUSCLE implemented in MEGA 7. The phylogenetic tree was generated using the software MEGA7 [67]. The tree is drawn to scale, with branch lengths in the same units as those of the evolutionary distances used to infer the phylogenetic tree. The evolutionary distances were computed using the Poisson correction method and are in the units of the number of amino acid substitutions per site. All positions containing gaps and missing data were eliminated. **(A)** Evolution history of all HsdS subunits detected in the ten *M. agalactiae* tested strains (strain name and subunit number as assigned in Figure 2). The optimal tree with the sum of branch length = 6.55524456 is shown. The analysis involved 27 amino acid sequences and there were a total of 36 positions in the final dataset. **(B)** Evolution history of all HsdM subunits detected in the ten *M. agalactiae* tested strains (strain name and subunit number as assigned in Figure 2). The optimal tree with the sum of branch length = 1.46693434 is shown. The analysis involved 18 amino acid sequences and there were a total of 418 positions in the final dataset. **(C)** Evolution history of all HsdR subunits detected in the ten *M. agalactiae tested* strains. The optimal tree with the sum of branch length = 1.41761699 is shown. The analysis involved 8 amino acid sequences and there were a total of 886 positions in the final dataset.

Figure S3 : Electropherograms regions corresponding to repeat tracts observed in genes encoding CpG and TypeIII Methyltransferases

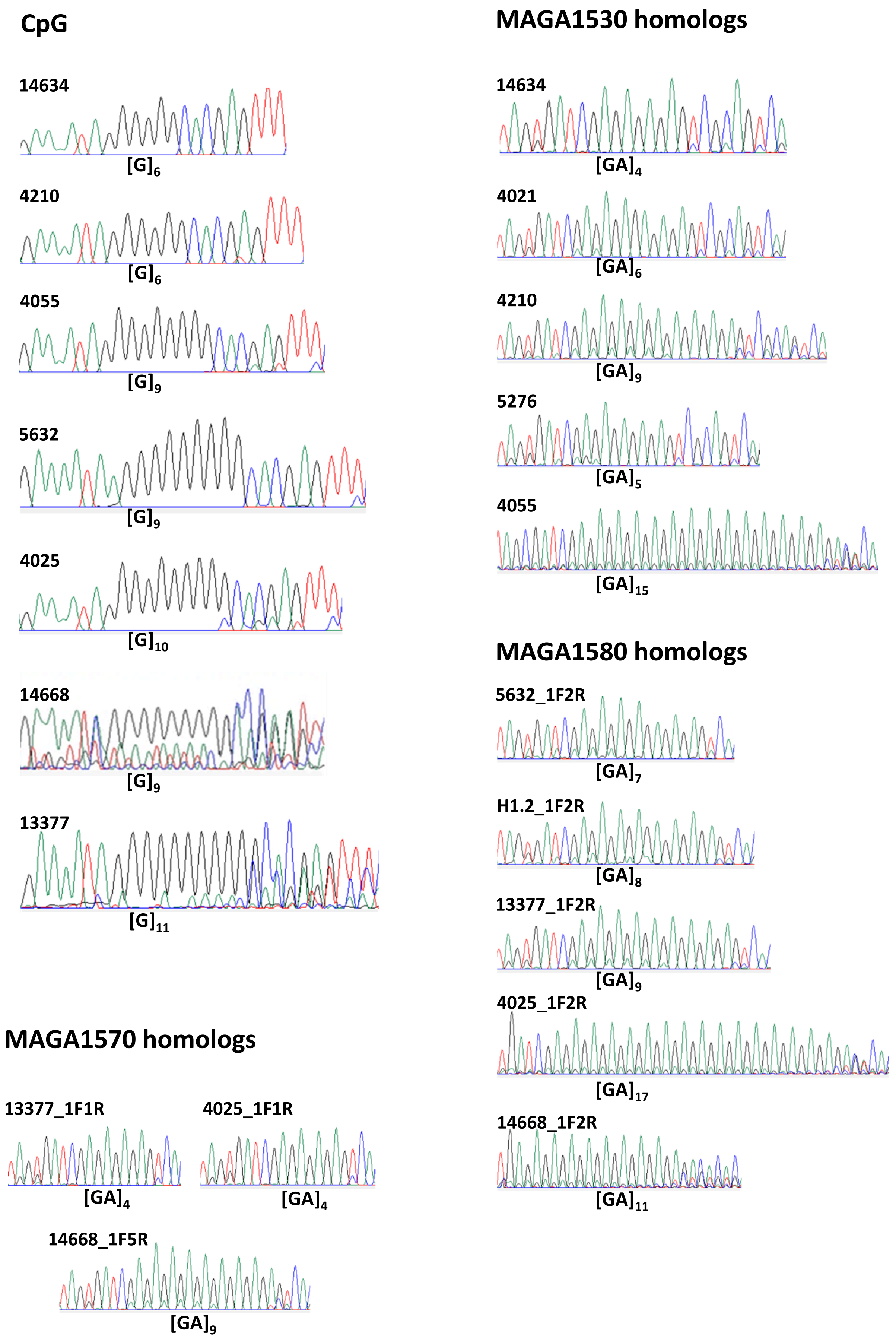

A

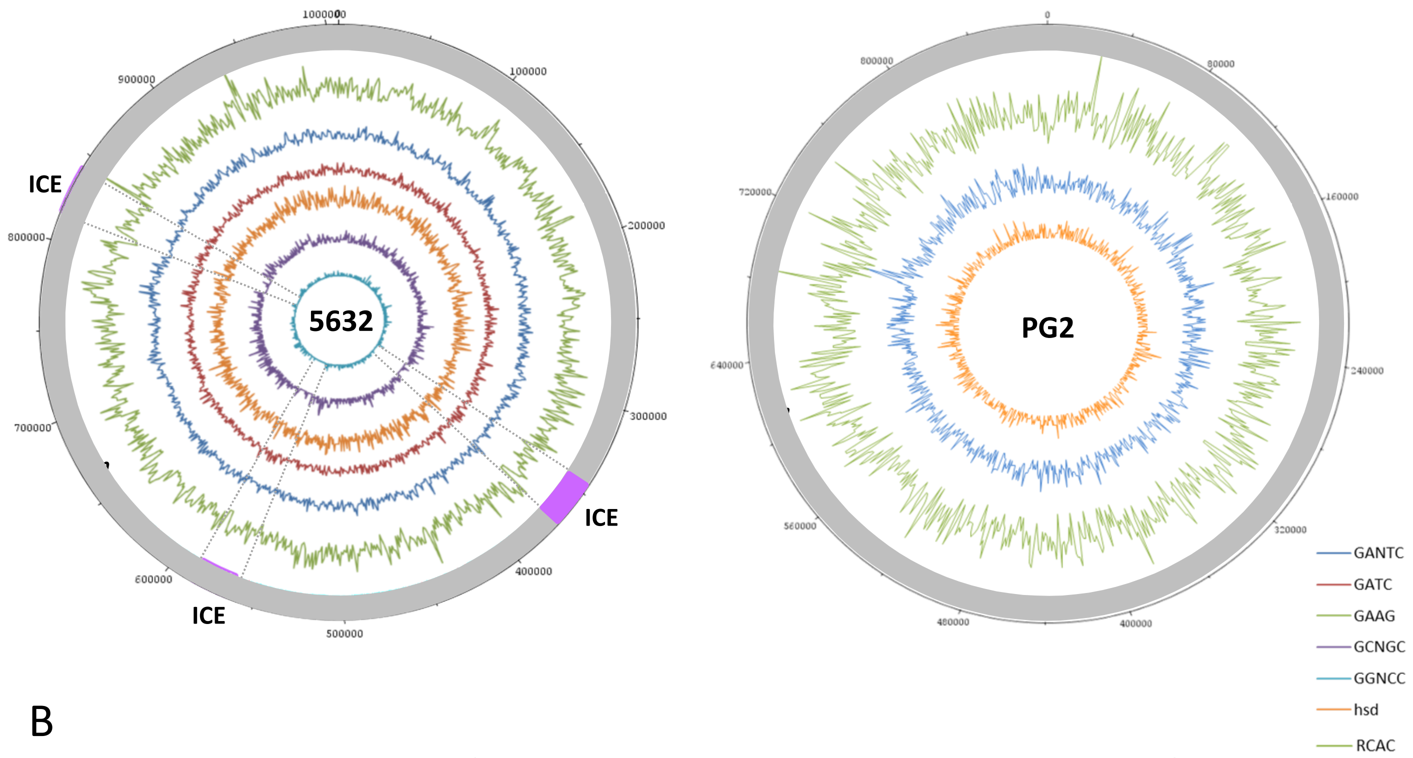

B

5632 DISTAMO analysis

	oriTer Biais	replichore bias
GAAG	-0,062	-1,184
GANTC	1,327	1,177
GATC	-1,998	-1,748
AYC(N)$_5$KTR	0,408	-1,301
GCNGC	1,028	-0,475
GGNCC	2,004	0,186

PG2 DISTAMO analysis

	oriTer Biais	replichore bias
RCAC	-0,063	0,065
GANTC	1,069	-0,108
AYC(N)$_6$TRG	0,214	1,209

Figure S4. Distribution of methylated motif along *M. agalactiae* 5632 and PG2 chromosomes. (A) Circular plot representing the distribution of methylated motifs calculated per 1-kb windows along the PG2 and 5632 chromosomes. From outer to inner circle for 5632 genome (grey circle) : GAAG (green), GANTC (dark blue), GATC (red), Type I methylated motifs (orange), GCNGC (purple), GGNCC (light blue). Integrative and conjugative elements (ICE) are represented by purple box in the grey outer circle representing the 5632 chromosome. From outer to inner circle for PG2 genome RCAC (green), GANTC (blue) and Type I methylated motifs (orange). **(B)** All methylated motifs distribution analyzed by Distamo [54] for 5632 and PG2 *M.agalactiae* chromosomes. The oriTer bias z-score corresponds to the bias of motif abundance on the *ori*C proximal half or the chromosome compared to the *ter* proximal half of the chromosome. A positive z-score above 2 indicates a significantly higher abundance of the motif on the *ori*C-proximal half. A z-score below -2 indicates a significantly higher abundance on the *ter*-proximal half. The replichore bias z-score corresponds to the motif abundance on the right replichore compared to the left replichore. A positive z-score above 2 indicates a significantly higher abundance of the motif on the right replichore. A z-score below -2 indicates a significantly higher abundance on the left replichore.

Table S1 : BLAST analysis against dcm REBASE database

Strain	GeneBank locus_tag	Homologs		Predicted motif	Size (nt)	Identity (aa)
**4210**	*J8A69_02160*	*M.Mag5632*	*MAGa4470*	*CG*	*426*	*96%*
	J8A69_02165	*M.Mag5632*	*MAGa4480*	*CG*	*747*	
**4021**	*J7890_02150*	*M.Mag5632*	*MAGa4470*	*CG*	*285*	*96%*
	J7890_02155	*M.Mag5632*	*MAGa4480*	*CG*	*771*	
**14634**	*J8A70_02150*	*M.MagPG2*	*MAG4250*	*CG*	*285*	*99%*
	J8A70_02155	*M.MagPG2*	*MAG4260*	*CG*	*771*	
**5276**	*J7891_02150*	*M.Mag5632*	*MAG4470*	*CG*	*285*	*96%*
	J7891_02155	*M.Mag5632*	*MAG4480*	*CG*	*771*	
**13377***	J7894_00205	M.MboH1	ORF688P	CCTC	1782	88%
	J7894_03755	M.Mag5632	MAGa4470	CG	285	98%
		M.Mag5632	MAGa4480	CG	771	
	J7894_00995	M.Mag5632	MAGa3950	GGNCC	1020	98%
	J7894_02115	M.MboP180	ORF173P	RCATGY	975	93%
**4025***	*J7889_02300*	*M.Mag5632*	*MAGa4470*	*CG*	*285*	*96%*
	J7889_02305	*M.Mag5632*	*MAGa4480*	*CG*	*771*	
	J7889_02035	M.Mag5632	MAGa3950	GGNCC	1020	98%
**14668***	*J8A71_02115*	M.Mag5632	MAGa4470	CG	*285*	98%
		M.Mag5632	MAGa4480	CG	*771*	
	J8A71_01800	M.Mag5632	MAG3950	GGNCC	1020	99%
**4055***	*J7892_02365*	M.Mag5632	MAGa4470	CG	*285*	89%
		M.Mag5632	MAGa4480	CG	*771*	
	J7892_02160	*M.Mag5632*	*MAG3950P*	*GGNCC*	*960*	*98%*

italic = pseudogene

*: strains chosen for BS sequencing because they contain dcm MTases other than the putative

Table S2. Primers used for PCR and sanger sequencing

	Name	Sequence 5' > 3'	Tm	Mtases and homologs	Strains
**Type III**	**1F_typeIII*#**	TACTATAAAACAGGATTATATAGAAAAAGCTAATGC	55°C		All strains included in this study
	**1R_type III**	TCTCCCGCTTGGGTTTAAGAT	60°C	MAGa1570	4025, 5632, 13377, 14668
	**2R_typeIII**	ATCTTCACCAGACCCTTCATTT	55°C	MAGa1580	4025, 5632, 13377
	**3R_typeIII**	CCTGAACCTCCGCCAGT	55°C	MAG1530	4021, 4210, 5276, 14634, PG2
	**4R_typeIII**	TGGCCCTCTTTTCTTAATTCATACC	55°C	MAG1530	4055
	**5R_typeIII**	GCTTAATGATGCTGGAGTATATGTTCT	55°C	MAG1580	14668
**CpG**	**1F_CpG#**	AGAAGTCAATAAAGCCTATTTTACAAGGATG	55°C	MAG4250-60 and MAGa 4470-80	All strains included in this study
	**1R_CpG**	AAAATTCTTTCAACTTCATAAAGCAATCC	55°C		

* : Universal Type III forward primer (F) used with all Type III reverse primers (R)

# : Primers used for Sanger sequencing and Fragment lenght analysis (FLA). For FLA these primers were labelled with 6-carboxyfluorescein (6-FAM) in 5’. For Type III, some strains possess 2 related genes that are organized as tandem and which sequences differ among strains (figure 4), thus the same reverse primer could not be used for all strains.

Table S3. Percentage of methylated TRD couples corresponding to Type I RM systems detected by SMRT sequencing compare to *in silico* motif abundance in 5632 and PG2 strains

5632

		TRD2			
		GTA	TTA	GTG	
TRD1	ATC	23% / 11%	31% / 52%	19% / 8%	36% / 40%
	ACC	9% / 6%	11% / 19%	7% / 4%	14% / 5%
		16% / 9%	21% / 39%	13% / 7%	

5632 Type I detected methylated sequences :

5'-ATC$(N)_5$GTA-3'

5'-ATC$(N)_5$TTA-3'

5'-ATC$(N)_5$GTG-3'

5'-ACC$(N)_5$GTA-3'

5'-ACC$(N)_5$TTA-3'

5'-ACC$(N)_5$GTG-3'

PG2

		TRD2		
		TAG	TGG	
TRD1	ATC	44% / 47%	22% / 26%	33% / 36%
	ACC	19% / 19%	16% / 8%	17% / 14%
		31% / 33%	19% / 17%	

PG2 Type I detected methylated sequences :

5'-ATC$(N)_6$TAG-3'

5'-ATC$(N)_6$TGG-3'

5'-ACC$(N)_6$TAG-3'

5'-ACC$(N)_6$TGG-3'

Abundance of detected TRD / Abundance of predicted TRD

number of detected TRD/total detected TRD / number predicted TRD/total *in silico* predicted TRDs

Table S4 : Methylated motif detected in PG2 and PG2 ICEA+ variants

						PG2 WT		PG2 ICEA+		nGenome
	**Detected motif**	**Consensus**	**Modification**	**Predicted motif**	**Assignated methylase**	**Fraction**	**nDetected**	**Fraction**	**nDetected**	**PG2**
**Type I**	AYC(N)$_6$RG	AYC(N)$_6$RG / CYA(N)$_6$GRT	m6A	-	MAG5660	85%	980	87%	990	1144
	CYA(N)$_6$GRT			-	MAG5730	74%	910	77%	879	1144
**Type II & III**	GANTC		m6A	GANTC	MAG6680	86%	4255	84%	4183	5004
	RCAC		m6A	-	MAG1530[#]	87%	7123	88%	7145	8081

Type III Mtase

Table S5. Fragment length analysis (FLA) of *M. agalactiae* Type III and CpG methyltransferases

	Reverse primer #	PCR theoretical size (bp)†	GA*	FLA: fragment 1		FLA: fragment 2		FLA: fragment 3	
				size (bp)	%	size (bp)	%	size (bp)	%
**Type III MAG1530**									
PG2	3R	666	4	662	100%	-	-	-	-
14634	3R	666	4	662	100%	-	-	-	-
4021	3R	670	6	666	100%	-	-	-	-
4210	3R	676	9	672	100%	-	-	-	-
5276	3R	668	5	664	100%	-	-	-	-
4055	4R	677	15	672	100%	-	-	-	--
**Type III MAGa1570**									
5632[a]	1R	655	4	651	100%	-	-	-	-
14668	5R	674	9	669	100%	-	-	-	-
**Type III MAGa1580**									
5632	2R	673	7	668	100%	-	-	-	-
5632H1-2	2R	675	8	671	100%	-	-	-	-
13377	2R	677	9	673	100%	-	-	-	-
4025	2R	693	17	687	34%	**688**	**66%**	-	-
14668	2R	678	11	670	24%	**672**	**41%**	674	35%

	PCR size (bp)	G*	FLA: fragment 1			FLA: fragment 2			FLA: fragment 3		
			size (bp)	%	G	size (bp)	%	G	size (bp)	%	G
**CpG**											
PG2[b]	301	6	294	nd	nd	295	29%	5	**296**	**71%**	**6**
14634 [b]	301	6	294	nd	nd	295	25%	5	**296**	**75%**	**6**
4055	304	9	297	7%	7	298	38%	8	**299**	**55%**	**9**
5632	305	9	298	nd	7	299	21%	8	**300**	**79%**	**9**
4025	305	10	298	nd	8	299	37%	9	**300**	**63%**	**10**
14668	304	9	297	15%	8	**298**	**51%**	**9**	299	34%	10
13377	306	11	299	7%	9	300	43%	10	**301**	50%	11

nd: pick heights under detection limit (<1000)

† Expected PCR product sizes were rounded. They differ from the size of FLA fragment defined by the Peak scanner software due to the fluorescent dye attached on the forward primer that may impaired migration depending on PCR sequences.

* Number of GAs or Gs in the polynucleotide tracts as defined based on Illumina and Sanger sequencing (see Figure S3)

a, b: Representative of group containing (a) 5632, 5632 H1-2, 4025 and 13377 strains and (b) PG2, 14634, 4021, 4210 and 5276 strains. Only one or two representatives were tested by FLA because of these groups having homologs with the same gene size and the same [GA] or [G] tract repeats (see Figure 4).

#: primers are described in Table S2, for CpG the same pair of oligonucleotides was used for all PCR products

Table S6 : CG abundance of *M. agalactiae* tested strains

	PG2	5632	4021	4025	4055	4210	5276	13377	14634	14668
**Total nt**	877439	944580	887230	1066370	1003709	884408	890157	1067640	905745	967428
Putative CpG MTase	-	+	-	+	-	-	-	+	-	+
Frequence CG#	0,01	0,01	0,01	0,01	0,01	0,01	0,01	0,01	0,01	0,01
Frequence C	0,15	0,15	0,15	0,15	0,15	0,15	0,15	0,15	0,15	0,15
Frequence G	0,14	0,15	0,14	0,15	0,14	0,14	0,14	0,15	0,14	0,15
CG abundundance (Fxy)*	0,49	0,46	0,48	0,49	0,50	0,49	0,49	0,49	0,49	0,49

nt = nucleotide

MTase = Methyltransferase

# number of nucleotides or dinucleotides present in the genome /total number of nucleotides

* Based on Goto et al. (2000) = Frequence of (CG) in the genome / (Frequence of (C) * Frequence of (G))

Table S7 : Over- and under-methylated genes based on DISTAMO analysis in *M. agalactiae* 5632 and PG2 stains

5632 DISTAMO analysis
Overrepresented genes
Underrepresented genes

	Genename	zScore	product	Mnemonic
GANTC	cdd	4,44	cytidine deaminase	MAGa1220
	ssb	2,75	single-stranded DNA-binding protein	MAGa4130
	vpmaE	-2,32	variable surface lipoprotein	MAGa5850
	vpmaE	-2,32	variable surface lipoprotein	MAGa8090
	vpmaW	-2,62	vpmaW: variable surface lipoprotein	vpmaW
GATC	vpmaE	3,39	variable surface lipoprotein	MAGa5850
	vpmaE	3,39	variable surface lipoprotein	MAGa8090
	rplU	3,17	50S ribosomal protein L21	MAGa6190
	MAGa4500	3,07	Lipoprot	MAGa4500
	MAGa2590	2,38	Lipoprot	MAGa2590
	MAGa3870	2,31	ATP synthase C chain	MAGa3870
	vpmaW	2,11	variable surface lipoprotein	
	MAGa1180	-2	Ribose 5 phosphate isomerase"	MAGa1180
	cmk	-2,09	Cytidylate kinase	MAGa8380
	prfA	-2,19	Peptide chain release factor 1	MAGa8520
	MAGa6690	-2,48	HP	MAGa6690
	prs	-2,52	ribose-phosphate pyrophosphokinase	MAGa1880
GAAG	MAGa6740	3,15	HP	MAGa6740
	MAGa5380	2,91	HP	MAGa5380
	rplL	2,77	50S ribosomal protein L7/L12"	rplL
	hit	2,57	HIT family protein	MAGa1990
	MAGa6460	2,24	GNAT family N-acetyltransferase	MAGa6460
	upp	2,19	uracil phosphoribosyltransferase	MAGa4120
	rpsI	-2,17	30S ribosomal protein S9	MAGa4780
	pknB	-2,19	Serine/threonine protein kinase	MAGa2120
	MAGa0310	-2,22	HP	MAGa0310
	MAGa7610	-2,82	CHP	MAGa7610
	MAGa1210	-3,08	CHP	MAGa1210
GCNGC	RlmH	3,02	23S rRNA (pseudouridine(1915)-N(3))-methyltransferase	MAGa1730
	MAGa1720	2,61	HP	MAGa1720
	rplK	2,25	50S ribosomal protein L11	MaGa0860
	MAga0570	2,08	HP	MAga0570
GGNCC	none			
$AYC(N)_5KTR$	none			

PG2 DISTAMO analysis
Overrepresented genes
Underrepresented genes

	Genename	zScore	product	Mnemonic
GANTC	MAG4350	4,3	Pseudogene of DNA processing protein (Smf)	MAG4350
	ssb	2,01	single-stranded DNA-binding protein	MAG3670
	infB	-2,01	Translation initiation factor IF-2	MAG6970
	vpmaW	-3,23	variable surface lipoprotein	
RCAC	MAG3140	4,35	HP	MAG3140
	MAG0845	2,96	Hypothetical protein, truncated in C terminal	MAG0845
	MAG1110	2,39	Ribose 5 phosphate isomerase	MAG1110
	pth	2,31	Peptidyl tRNA hydrolase	MAG7470
	MAG6865	2,3	HP	MAG6865
	MAG4420	2,13	HP	MAG4420
	hit	2,11	HIT like protein (Cell cycle regulation)	MAG1990
	MAG2790	2,08	CHP	MAG2790
	rplA	-2,09	50S ribosomal protein L1	MAG0810
	MAG5670	-2,25	CHP truncated in N term	MAG5670
$AYC(N)_6TRG$	MAG4330	-2,24	HP lipoprotein	ompH

Table S8. Mobilome of the ten sequenced *M. agalactiae* strains

Strain	Mnemonic	Contig	Gene name	Function
**PG2**	MAG_3410	complete genome		Mobile element protein
	MAG_6150	complete genome		Mobile element protein
	MAG_7110	complete genome		Integrase-recombinase
	MAG_5690	complete genome		Integrase
	MAG6440	complete genome		Putative prophage protein (ps3)
	MAG6440b	complete genome		Putative prophage protein (ps3) second copy
	MAG_2380	complete genome	cds17	
	MAG_3360	complete genome	cds22	
	MAG_3610	complete genome	cdsB	
	MAG_3860	complete genome	cds22	
	MAG_3890	complete genome	cds19	
	MAG_3910	complete genome	cds17	
	MAG_3920	complete genome	cds17	
	MAG_3940	complete genome	cds16	
	MAG_3980	complete genome	cdsC	
	MAG_3990	complete genome	cds12	
	MAG_4040	complete genome	cds5	
	MAG_4780	complete genome	cds17	
**4210**	peg.101	contig_1		Mobile element protein
	peg.713	contig_1		Mobile element protein
	peg.45	contig_1		Phage integrase
	peg.203	contig_1		Integrase-recombinase
	peg.631	contig_1		transposase
	peg.632	contig_1		transposase
	peg.133	contig_1		Putative prophage protein (ps3)
	peg.445	contig_1	cds17	ICE element
	peg.516	contig_1	cds17	ICE element
	peg.625	contig_1	cds22	ICE element
	peg.626	contig_1	cds22	ICE element
	peg.657	contig_1	cdsB	ICE element
	peg.685	contig_1	cds22	ICE element
	peg.686	contig_1	cds22	ICE element
	peg.689	contig_1	cds19	ICE element
	peg.691	contig_1	cds17	ICE element
	peg.692	contig_1	cds17	ICE element
	peg.693	contig_1	cds17	ICE element
	peg.694	contig_1	cds16	ICE element
	peg.695	contig_1	cds15	ICE element
	peg.699	contig_1	cdsC	ICE element
	peg.700	contig_1	cds12	ICE element
	peg.706	contig_1	cds5	ICE element
	peg.707	contig_1	cds5	ICE element
	peg.785	contig_1	cds17	ICE element
**4021**	peg.1	contig_1		Mobile element protein
	peg.2	contig_1		Mobile element protein
	peg.283	contig_2		Mobile element protein
	peg.534	contig_2		Mobile element protein
	peg.140	contig_1		Integrase-recombinase
	peg.170	contig_1		Integrase-recombinase
	peg.340	contig_2		Phage integrase
	peg.1035	contig_5		transposase
	peg.1036	contig_5		transposase
	peg.252	contig_2		Putative prophage protein (ps3)
	peg.458	contig_2	cds17	ICE element
	peg.542	contig_2	cds5	ICE element
	peg.548	contig_2	cds12	ICE element
	peg.549	contig_2	cdsC	ICE element
	peg.554	contig_2	cds15	ICE element
	peg.555	contig_2	cds16	ICE element
	peg.556	contig_2	cds17	ICE element
	peg.557	contig_2	cds17	ICE element
	peg.559	contig_2	cds17	ICE element

	peg.560	contig_2	cds17	ICE element
	peg.562	contig_2	cds19	ICE element
	peg.565	contig_2	cds22	ICE element
	peg.600	contig_2	cdsB	ICE element
	peg.693	contig_3	cds17	ICE element
	peg.1028	contig_5	cds22	ICE element
	peg.1029	contig_5	cds22	ICE element
	peg.1060	contig_5	cdsB	ICE element
**5276**	peg.367	contig_1		Mobile element protein
	peg.660	contig_1		Mobile element protein
	peg.820	contig_3		Mobile element protein
	peg.821	contig_3		Mobile element protein
	peg.822	contig_3		Mobile element protein
	peg.823	contig_3		Mobile element protein
	peg.608	contig_1		Integrase
	peg.781	contig_2		Integrase-recombinase
	peg.691	contig_1		Putative prophage protein (ps3)
	peg.714	contig_2		Putative prophage protein (ps3)
	peg.715	contig_2		Putative prophage protein (ps3)
	peg.258	contig_1	cds17	ICE element
	peg.361	contig_1	cds22	ICE element
	peg.362	contig_1	cds22	ICE element
	peg.389	contig_1	cdsB	ICE element
	peg.415	contig_1	cds22	ICE element
	peg.418	contig_1	cds19	ICE element
	peg.420	contig_1	cds17	ICE element
	peg.422	contig_1	cds17	ICE element
	peg.423	contig_1	cds16	ICE element
	peg.424	contig_1	cds15	ICE element
	peg.428	contig_1	cdsC	ICE element
	**peg.429**	contig_1	cds12	ICE element
	**peg.435**	contig_1	cds5	ICE element
	peg.513	contig_1	cds17	ICE element
**14634**	peg.134	contig_1		Mobile element protein
	peg.927	contig_3		Mobile element protein
	peg.953	contig_3		Mobile element protein
	peg.688	contig_1		Integrase-recombinase
	peg.689	contig_1		Integrase-recombinase
	peg.744	contig_2		Phage integrase
	peg.828	contig_3		Integrase-recombinase
	peg.220	contig_1		transposase
	peg.893	contig_3		Putative prophage protein (ps3)
	peg.57	contig_1	cds17	ICE element
	peg.141	contig_1	cds5	ICE element
	peg.147	contig_1	cds12	ICE element
	peg.148	contig_1	cdsC	ICE element
	peg.153	contig_1	cds15	ICE element
	peg.154	contig_1	cds16	ICE element
	peg.155	contig_1	cds17	ICE element
	peg.156	contig_1	cds17	ICE element
	peg.158	contig_1	cds17	ICE element
	peg.161	contig_1	cds19	ICE element
	peg.164	contig_1	cds22	ICE element
	peg.165	contig_1	cds22	ICE element
	peg.198	contig_1	cdsB	ICE element
	peg.226	contig_1	cds22	ICE element
	peg.351	contig_1	cds17	ICE element
	peg.425	contig_1	cds17	ICE element
	peg.569	contig_1	cdsE	ICE element
**5632**	MAGa1590	complete genome		Mobile element protein
	MAGa5640	complete genome		Mobile element protein
	MAGa7100	complete genome		Mobile element protein
	MAGa7110	complete genome		Mobile element protein
	MAGa7420	complete genome		Mobile element protein
	MAGa5880	complete genome		Integrase-recombinase
	MAGa6320	complete genome		Phage integrase

MAGa6690	complete genome		Transposase
MAGa7400	complete genome		Putative prophage protein (ps3)
MAGa2700	complete genome	cdsH	ICE element
MAGa2980	complete genome	cds1	ICE element
MAGa2990	complete genome	cdsA	ICE element
MAGa3000	complete genome	cds12	ICE element
MAGa3010	complete genome	cds11	ICE element
MAGa3020	complete genome	cds11	ICE element
MAGa3030	complete genome	cdsB	ICE element
MAGa3040	complete genome	cdsC	ICE element
MAGa3050	complete genome	cdsD	ICE element
MAGa3060	complete genome	cds5	ICE element
MAGa3070	complete genome	cds7	ICE element
MAGa3080	complete genome	cds13	ICE element
MAGa3090	complete genome	cds15	ICE element
MAGa3100	complete genome	cds16	ICE element
MAGa3110	complete genome	cds16	ICE element
MAGa3130	complete genome	cds17	ICE element
MAGa3140	complete genome	cds19	ICE element
MAGa3150	complete genome	cdsE	ICE element
MAGa3160	complete genome	cds14	ICE element
MAGa3170	complete genome	cdsF	ICE element
MAGa3190	complete genome	cdsG	ICE element
MAGa3200	complete genome	cdsH	ICE element
MAGa3210	complete genome	cdsG	ICE element
MAGa3220	complete genome	cds22	ICE element
MAGa3670	complete genome	cds22	ICE element
MAGa3680	complete genome	cds22	ICE element
MAGa4000	complete genome	cds22	ICE element
MAGa4040	complete genome	cds5	ICE element
MAGa4070	complete genome	cdsB	ICE element
MAGa4850	complete genome	cds1	ICE element
MAGa4860	complete genome	cdsA	ICE element
MAGa4870	complete genome	cds12	ICE element
MAGa4880	complete genome	cds11	ICE element
MAGa4890	complete genome	cdsB	ICE element
MAGa4900	complete genome	cdsC	ICE element
MAGa4910	complete genome	cdsD	ICE element
MAGa4920	complete genome	cds5	ICE element
MAGa4930	complete genome	cds7	ICE element
MAGa4940	complete genome	cds13	ICE element
MAGa4950	complete genome	cds15	ICE element
MAGa4960	complete genome	cds16	ICE element
MAGa4980	complete genome	cds17	ICE element
MAGa4990	complete genome	cds19	ICE element
MAGa5000	complete genome	cdsE	ICE element
MAGa5010	complete genome	cds14	ICE element
MAGa5020	complete genome	cdsF	ICE element
MAGa5040	complete genome	cdsG	ICE element
MAGa5050	complete genome	cdsH	ICE element
MAGa5060	complete genome	cds22	ICE element
MAGa5250	complete genome	cds17	ICE element
MAGa6880	complete genome	cds22	ICE element
MAGa6890	complete genome	cdsG	ICE element
MAGa6900	complete genome	cdsH	ICE element
MAGa6910	complete genome	cdsG	ICE element
MAGa6930	complete genome	cdsF	ICE element
MAGa6940	complete genome	cds14	ICE element
MAGa6950	complete genome	cdsE	ICE element
MAGa6960	complete genome	cds19	ICE element
MAGa6970	complete genome	cds17	ICE element
MAGa6990	complete genome	cds16	ICE element
MAGa7000	complete genome	cds15	ICE element
MAGa7010	complete genome	cds13	ICE element

	MAGa7020	complete genome	cds7	ICE element
	MAGa7030	complete genome	cds5	ICE element
	MAGa7040	complete genome	cdsD	ICE element
	MAGa7050	complete genome	cdsC	ICE element
	MAGa7060	complete genome	cdsB	ICE element
	MAGa7070	complete genome	cds11	ICE element
	MAGa7080	complete genome	cds12	ICE element
	MAGa7090	complete genome	cdsA	ICE element
	MAGa7100	complete genome	cds1	ICE element
**13377**	peg.1	contig_1		Mobile element protein
	peg.2	contig_1		Mobile element protein
	peg.3	contig_1		Mobile element protein
	peg.55	contig_1		Mobile element protein
	peg.130	contig_1		Mobile element protein
	peg.131	contig_1		Mobile element protein
	peg.211	contig_15		Mobile element protein
	peg.228	contig_15		Mobile element protein
	peg.231	contig_15		Mobile element protein
	peg.243	contig_15		Mobile element protein
	peg.253	contig_15		Mobile element protein
	peg.254	contig_15		Mobile element protein
	peg.280	contig_16		Mobile element protein
	peg.281	contig_16		Mobile element protein
	peg.282	contig_16		Mobile element protein
	peg.290	contig_16		Mobile element protein
	peg.326	contig_17		Mobile element protein
	peg.343	contig_17		Mobile element protein
	peg.485	contig_5		Mobile element protein
	peg.626	contig_7		Mobile element protein
	peg.645	contig_7		Mobile element protein
	peg.676	contig_7		Mobile element protein
	peg.712	contig_8		Mobile element protein
	peg.795	contig_8		Mobile element protein
	peg.848	contig_8		Mobile element protein
	peg.863	contig_8		Mobile element protein
	peg.878	contig_8		Mobile element protein
	peg.879	contig_8		Mobile element protein
	peg.1054	contig_8		Mobile element protein
	peg.891	contig_8		Integrase-recombinase
	peg.223	contig_15		transposase
	peg.63	contig_1		Putative prophage protein (ps3)
	peg.255	contig_15		Phage helicase
	peg.262	contig_15		phage DNA polymerase
	peg.264	contig_15		DNA primase, phage associated
	peg.265	contig_15		Phage DNA primase
	peg.696	contig_7		phage DNA polymerase
	peg.697	contig_7		DNA primase, phage associated
	peg.4	contig_1	cds22	ICE element
	peg.5	contig_1	cds7	ICE element
	peg.6	contig_1	cds5	ICE element
	peg.7	contig_1	cds5	ICE element
	peg.8	contig_1	cds5	ICE element
	peg.9	contig_1	cdsD	ICE element
	peg.10	contig_1	cdsD	ICE element
	peg.12	contig_1	cdsC	ICE element
	peg.13	contig_1	cdsC	ICE element
	peg.14	contig_1	cdsB	ICE element
	peg.15	contig_1	cds11	ICE element
	peg.16	contig_1	cds11	ICE element
	peg.17	contig_1	cds12	ICE element
	peg.18	contig_1	cdsA	ICE element
	peg.20	contig_1	cds1	ICE element
	peg.21	contig_1	cds1	ICE element
	peg.205	contig_15	cds22	ICE element
	peg.206	contig_15	cds22	ICE element
	peg.271	contig_16	cdsA	ICE element

	peg.272	contig_16	cds12	ICE element
	peg.273	contig_16	cds11	ICE element
	peg.274	contig_16	cdsB	ICE element
	peg.275	contig_16	cdsC	ICE element
	peg.276	contig_16	cdsD	ICE element
	peg.277	contig_16	cds5	ICE element
	peg.278	contig_16	cds7	ICE element
	peg.279	contig_16	cds22	ICE element
	peg.320	contig_17	cds5	ICE element
	peg.321	contig_17	cds5	ICE element
	peg.327	contig_17	cdsB	ICE element
	peg.345	contig_18	cds19	ICE element
	peg.346	contig_18	cds19	ICE element
	peg.347	contig_18	cds17	ICE element
	peg.348	contig_18	cds17	ICE element
	peg.349	contig_18	cds16	ICE element
	peg.350	contig_18	cds17	ICE element
	peg.352	contig_18	cds19	ICE element
	peg.354	contig_18	cds22	ICE element
	peg.371	contig_20	cdsH	ICE element
	peg.402	contig_5	cds1	ICE element
	peg.404	contig_5	cdsA	ICE element
	peg.405	contig_5	cds12	ICE element
	peg.406	contig_5	cds11	ICE element
	peg.407	contig_5	cdsB	ICE element
	peg.408	contig_5	cdsC	ICE element
	peg.409	contig_5	cdsD	ICE element
	peg.410	contig_5	cds5	ICE element
	peg.411	contig_5	cds7	ICE element
	peg.413	contig_5	cds15	ICE element
	peg.414	contig_5	cds16	ICE element
	peg.415	contig_5	cds22	ICE element
	peg.492	contig_5	cdsF	ICE element
	peg.494	contig_5	cdsG	ICE element
	peg.495	contig_5	cdsG	ICE element
	peg.496	contig_5	cdsH	ICE element
	peg.497	contig_5	cdsG	ICE element
	peg.498	contig_5	cds22	ICE element
	peg.507	contig_5	cdsH	ICE element
	peg.585	contig_5	cds17	ICE element
	peg.592	contig_5	cds1	ICE element
	peg.594	contig_5	cds5	ICE element
	peg.597	contig_5	cdsC	ICE element
	peg.599	contig_5	cds12	ICE element
	peg.603	contig_5	cds15	ICE element
	peg.604	contig_5	cds22	ICE element
	peg.605	contig_5	cdsG	ICE element
	peg.606	contig_5	cdsH	ICE element
	peg.607	contig_5	cdsG	ICE element
	peg.609	contig_5	cdsF	ICE element
	peg.613	contig_5	cds14	ICE element
	peg.614	contig_5	cdsE	ICE element
	peg.615	contig_5	cds19	ICE element
	peg.616	contig_5	cds17	ICE element
	peg.618	contig_5	cds16	ICE element
	peg.790	contig_8	cds17	ICE element
**4025**	peg.171	contig_1		Mobile element protein
	peg.172	contig_1		Mobile element protein
	peg.194	contig_12		Mobile element protein
	peg.197	contig_12		Mobile element protein
	peg.198	contig_12		Mobile element protein
	peg.199	contig_12		Mobile element protein
	peg.254	contig_12		Mobile element protein
	peg.255	contig_12		Mobile element protein
	peg.259	contig_12		Mobile element protein
	peg.263	contig_12		Mobile element protein

peg.311	contig_12		Mobile element protein
peg.312	contig_12		Mobile element protein
peg.315	contig_12		Mobile element protein
peg.323	contig_12		Mobile element protein
peg.363	contig_12		Mobile element protein
peg.388	contig_12		Mobile element protein
peg.396	contig_12		Mobile element protein
peg.400	contig_12		Mobile element protein
peg.401	contig_12		Mobile element protein
peg.402	contig_12		Mobile element protein
peg.445	contig_12		Mobile element protein
peg.446	contig_12		Mobile element protein
peg.448	contig_12		Mobile element protein
peg.488	contig_12		Mobile element protein
peg.506	contig_12		Mobile element protein
peg.507	contig_12		Mobile element protein
peg.509	contig_12		Mobile element protein
peg.547	contig_12		Mobile element protein
peg.565	contig_12		Mobile element protein
peg.573	contig_12		Mobile element protein
peg.581	contig_12		Mobile element protein
peg.616	contig_12		Mobile element protein
peg.622	contig_12		Mobile element protein
peg.623	contig_12		Mobile element protein
peg.645	contig_12		Mobile element protein
peg.742	contig_14		Mobile element protein
peg.752	contig_2		Mobile element protein
peg.796	contig_2		Mobile element protein
peg.798	contig_2		Mobile element protein
peg.799	contig_2		Mobile element protein
peg.800	contig_2		Mobile element protein
peg.803	contig_2		Mobile element protein
peg.816	contig_2		Mobile element protein
peg.900	contig_4		Mobile element protein
peg.931	contig_4		Mobile element protein
peg.932	contig_4		Mobile element protein
peg.933	contig_4		Mobile element protein
peg.494	contig_12		Integrase
peg.705	contig_14		Integrase-recombinase
peg.535	contig_12		Transposase
peg.603	contig_12		Putative prophage protein (ps3)
peg.153	contig_1	cds1	ICE element
peg.155	contig_1	cds5	ICE element
peg.159	contig_1	cds12	ICE element
peg.161	contig_1	cds12	ICE element
peg.164	contig_1	cds5	ICE element
peg.166	contig_1	cds1	ICE element
peg.278	contig_12	cds22	ICE element
peg.283	contig_12	cds5	ICE element
peg.287	contig_12	cdsB	ICE element
peg.387	contig_12	cds17	ICE element
**peg.543**	contig_12	cds22	ICE element
**peg.548**	contig_12	cds19	ICE element
**peg.550**	contig_12	cds17	ICE element
**peg.551**	contig_12	cds16	ICE element
**peg.552**	contig_12	cds15	ICE element
**peg.556**	contig_12	cds12	ICE element
**peg.557**	contig_12	cdsC	ICE element
**peg.558**	contig_12	cds12	ICE element
**peg.561**	contig_12	cds5	ICE element
**peg.563**	contig_12	cds1	ICE element
peg.583	contig_12	cdsH	ICE element
peg.584	contig_12	cdsH	ICE element
peg.749	contig_2	cds19	ICE element
peg.750	contig_2	cds19	ICE element
peg.756	contig_2	cds22	ICE element

	peg.786	contig_2	cdsH	ICE element
	peg.863	contig_2	cds17	ICE element
	peg.878	contig_2	cdsH	ICE element
	peg.907	contig_4	cds1	ICE element
	peg.911	contig_4	cds5	ICE element
	peg.912	contig_4	cds5	ICE element
	peg.913	contig_4	cds5	ICE element
	peg.920	contig_4	cds5	ICE element
	peg.921	contig_4	cds5	ICE element
	peg.925	contig_4	cds1	ICE element
	peg.926	contig_4	cds1	ICE element
	peg.958	contig_8	cds1	ICE element
	peg.963	contig_8	cds5	ICE element
	peg.964	contig_8	cds5	ICE element
	peg.970	contig_8	cds5	ICE element
	peg.971	contig_8	cds5	ICE element
	peg.972	contig_8	cds5	ICE element
	peg.973	contig_8	cds5	ICE element
	peg.974	contig_8	cds5	ICE element
	peg.980	contig_8	cds1	ICE element
	peg.981	contig_8	cds1	ICE element
**14668**	peg.258	contig_1		Mobile element protein
	peg.259	contig_1		Mobile element protein
	peg.433	contig_1		Mobile element protein
	peg.614	contig_1		Mobile element protein
	peg.615	contig_1		Mobile element protein
	peg.792	contig_2		Mobile element protein
	peg.657	contig_1		integrase-recombinase protein
	peg.685	contig_1		Integrase-recombinase
	peg.626	contig_1		DNA helicase, phage-associated
	peg.636	contig_1		DNA primase, phage associated # P4-type
	peg.650	contig_1		Phage tail fiber protein
	peg.653	contig_1		Phage portal protein
	peg.654	contig_1		Phage terminase, large subunit
	peg.768	contig_2		Putative prophage protein (ps3)
	peg.242	contig_1	cds17	ICE element
	peg.330	contig_1	cds22	ICE element
	peg.331	contig_1	cds22	ICE element
	peg.361	contig_1	cds22	ICE element
	peg.365	contig_1	cds19	ICE element
	peg.367	contig_1	cds17	ICE element
	peg.368	contig_1	cds16	ICE element
	peg.369	contig_1	cds15	ICE element
	peg.373	contig_1	cdsC	ICE element
	peg.374	contig_1	cdsC	ICE element
	peg.375	contig_1	cds12	ICE element
	peg.377	contig_1	cdsA	ICE element
	peg.380	contig_1	cds5	ICE element
	peg.383	contig_1	cdsB	ICE element
	peg.479	contig_1	cds17	ICE element
	peg.789	contig_2	cdsH	ICE element
	peg.790	contig_2	cdsH	ICE element
**4055**	peg.60	contig_1		Mobile element protein
	peg.61	contig_1		Mobile element protein
	peg.64	contig_1		Mobile element protein
	peg.65	contig_1		Mobile element protein
	peg.68	contig_1		Mobile element protein
	peg.178	contig_1		Mobile element protein
	peg.403	contig_1		Mobile element protein
	peg.405	contig_1		Mobile element protein
	peg.699	contig_1		Mobile element protein
	peg.702	contig_1		Mobile element protein
	peg.708	contig_1		Mobile element protein
	peg.816	contig_1		Mobile element protein
	peg.817	contig_1		Mobile element protein
	peg.869	contig_1		Mobile element protein

peg.874	contig_1		Mobile element protein
peg.931	contig_1		Mobile element protein
peg.980	contig_1		Mobile element protein
peg.983	contig_1		Mobile element protein
peg.987	contig_1		Mobile element protein
peg.1028	contig_1		Mobile element protein
peg.1032	contig_1		Mobile element protein
peg.1130	contig_1		Mobile element protein
peg.1131	contig_1		Mobile element protein
peg.1133	contig_1		Mobile element protein
peg.1322	contig_1		Mobile element protein
peg.1323	contig_1		Mobile element protein
peg.1334	contig_1		Mobile element protein
peg.1394	contig_1		Mobile element protein
peg.1396	contig_1		Mobile element protein
peg.1533	contig_1		Mobile element protein
peg.1535	contig_1		Mobile element protein
peg.1548	contig_1		Mobile element protein
peg.1570	contig_1		Mobile element protein
peg.1608	contig_1		Mobile element protein
peg.1626	contig_1		Mobile element protein
peg.1646	contig_1		Mobile element protein
peg.1671	contig_1		Mobile element protein
peg.1740	contig_1		Mobile element protein
peg.1748	contig_1		Mobile element protein
peg.1750	contig_1		Mobile element protein
peg.1772	contig_1		Mobile element protein
peg.1782	contig_1		Mobile element protein
peg.1866	contig_1		Mobile element protein
peg.1891	contig_1		Mobile element protein
peg.1895	contig_1		Mobile element protein
peg.1952	contig_1		Mobile element protein
peg.1956	contig_1		Mobile element protein
peg.1959	contig_1		Mobile element protein
peg.1963	contig_1		Mobile element protein
peg.1964	contig_1		Mobile element protein
peg.1966	contig_1		Mobile element protein
peg.1969	contig_1		Mobile element protein
peg.1975	contig_1		Mobile element protein
peg.1976	contig_1		Mobile element protein
peg.1978	contig_1		Mobile element protein
peg.2352	contig_1		Mobile element protein
peg.2551	contig_1		Mobile element protein
peg.2731	contig_1		Mobile element protein
peg.2732	contig_1		Mobile element protein
peg.2735	contig_1		Mobile element protein
peg.2749	contig_1		Mobile element protein
peg.2808	contig_1		Mobile element protein
peg.2810	contig_1		Mobile element protein
peg.2866	contig_1		Mobile element protein
peg.2869	contig_1		Mobile element protein
peg.2905	contig_1		Mobile element protein
peg.1791	contig_1		integrase-recombinase protein
peg.2399	contig_1		Transposase ISMmy1I
peg.497	contig_1		Putative prophage protein (ps3)
peg.498	contig_1		Putative prophage protein (ps3)
peg.496	contig_1	cds14	ICE element
peg.1418	contig_1	cds5	ICE element

Table S10. BLASTP results for *M. agalactiae* active methyltransferases against bacteria other than the Mollicutes class

Methyltransferases *	Description	Scientific Name	Max Score	Total Score	Query Cover	E value	Per. Ident	Acc. Len	Accession
MAGa2700	Dam family site-specific DNA-(adenine-N6)-methyltransferase [Solobacterium sp.]	Solobacterium sp.	338	338	96%	1,00E-113	59.34%	273	MBF1103133.1
	Dam family site-specific DNA-(adenine-N6)-methyltransferase [Solobacterium sp.]	Solobacterium sp.	336	336	96%	5,00E-113	59.34%	273	MBF1078290.1
	Dam family site-specific DNA-(adenine-N6)-methyltransferase [Solobacterium sp.]	Solobacterium sp.	335	335	96%	1,00E-112	58.97%	273	MBF1086133.1
	DNA adenine methylase [Macrococcus caseolyticus]	Macrococcus caseolyticus	332	332	96%	2,00E-111	59.11%	269	RKO15992.1
	DNA adenine methylase [Macrococcus sp. IME1552]	Macrococcus sp. IME1552	331	331	96%	8,00E-111	59.11%	269	WP_096077300.1
	DNA adenine methylase [Macrococcus caseolyticus]	Macrococcus caseolyticus	325	650	96%	1,00E-108	58.36%	269	WP_099483891.1
	Dam family site-specific DNA-(adenine-N6)-methyltransferase [Macrococcus canis]	Macrococcus canis	325	325	96%	2,00E-108	57.99%	269	WP_164941655.1
	DNA adenine methylase [Megamonas hypermegale]	Megamonas hypermegale	324	324	96%	4,00E-108	58.30%	276	WP_027889300.1
	Modification methylase DpnIIA [Megamonas hypermegale]	Megamonas hypermegale	324	324	96%	4,00E-108	58.30%	278	SNU95784.1
	MULTISPECIES: Dam family site-specific DNA-(adenine-N6)-methyltransferase [Staphylococcus]	Staphylococcus	323	323	96%	7,00E-108	56.88%	269	WP_071560813.1
MAGa7650	site-specific DNA-methyltransferase [Clostridia bacterium]	Clostridia bacterium	469	469	99%	3,00E-162	61.44%	376	NLO90117.1
	site-specific DNA-methyltransferase [Treponema vincentii]	Treponema vincentii	463	463	98%	6,00E-160	61.02%	372	WP_006187946.1
	site-specific DNA-methyltransferase [Treponema vincentii]	Treponema vincentii	462	462	98%	1,00E-159	61.29%	372	WP_016518474.1
	site-specific DNA-methyltransferase [Campylobacter curvus]	Campylobacter curvus	459	459	96%	2,00E-158	62.47%	371	WP_169783906.1
	site-specific DNA-methyltransferase [Clostridium isatidis]	Clostridium isatidis	458	458	93%	4,00E-158	62.61%	359	WP_119866576.1
	site-specific DNA-methyltransferase [Campylobacter concisus]	Campylobacter concisus	455	455	97%	1,00E-156	60.60%	369	WP_107832996.1
	MULTISPECIES: site-specific DNA-methyltransferase [Campylobacter]	Campylobacter	454	454	97%	2,00E-156	60.87%	369	WP_009294648.1
	MULTISPECIES: site-specific DNA-methyltransferase [unclassified Campylobacter]	unclassified Campylobacter	454	454	97%	2,00E-156	59.67%	369	WP_086237322.1
	site-specific DNA-methyltransferase [Marinitoga sp. 1138]	Marinitoga sp. 1138	453	453	97%	4,00E-156	60.48%	372	WP_175418024.1
	site-specific DNA-methyltransferase [Campylobacter hyointestinalis]	Campylobacter hyointestinalis	452	452	97%	1,00E-155	59.40%	370	WP_147499997.1
MAGa4250	DNA cytosine methyltransferase [Fusobacterium sp. CM21]	Fusobacterium sp. CM21	469	469	100%	1,00E-163	70.79%	351	WP_032841141.1
	M.Fnu4HI [Fusobacterium nucleatum]	Fusobacterium nucleatum	469	469	100%	1,00E-163	70.48%	351	ADX97301.1
	DNA (cytosine-5-)-methyltransferase [Parvimonas sp. S3374]	Parvimonas sp. S3374	458	458	99%	2,00E-159	71.34%	347	WP_201275014.1
	DNA cytosine methyltransferase [Parvimonas sp. S3374]	Parvimonas sp. S3374	458	458	99%	3,00E-159	71.34%	346	MBK1467862.1
	DNA cytosine methyltransferase [Bacillus toyonensis]	Bacillus toyonensis	456	456	99%	3,00E-159	67.52%	315	WP_098944381.1
	DNA cytosine methyltransferase [Sphingobium sp. TCM1]	Sphingobium sp. TCM1	454	454	99%	3,00E-158	67.52%	315	WP_066863026.1
	DNA cytosine methyltransferase [Bacillus mycoides]	Bacillus mycoides	452	452	99%	2,00E-157	67.52%	315	WP_016127586.1
	RecName: Full=Modification methylase Bsp6I; Short=M.Bsp6I; AltName: Full=Cytosine-specific methyltransfe	Bacillus sp. RFL6	450	450	99%	9,00E-157	66.56%	315	P43420.1
	DNA (cytosine-5-)-methyltransferase [Hazenella sp. IB182357]	Hazenella sp. IB182357	448	448	99%	1,00E-155	67.20%	324	WP_191142604.1
	TPA: DNA cytosine methyltransferase [Bacilli bacterium]	Bacilli bacterium	441	441	99%	1,00E-152	64.76%	332	HHU19175.1
MAGa3950	DNA (cytosine-5-)-methyltransferase [Epulopiscium sp. Nele67-Bin005]	Epulopiscium sp. Nele67-Bin005	510	510	98%	1,00E-179	70.57%	345	OON95436.1
	DNA (cytosine-5-)-methyltransferase [Bisgaard taxon 44 str. B96_4]	Bisgaard taxon 44 str. B96_4	501	501	96%	3,00E-176	73.62%	341	RIY34047.1
	DNA (cytosine-5-)-methyltransferase [Campylobacter sp. RM15925]	Campylobacter sp. RM15925	499	499	98%	4,00E-175	73.05%	343	WP_169941814.1
	DNA (cytosine-5-)-methyltransferase [Fusobacterium periodonticum 2_1_31]	Fusobacterium periodonticum 2_1	495	495	97%	2,00E-173	70.18%	361	KGE62123.1
	DNA cytosine methyltransferase [Fusobacterium nucleatum]	Fusobacterium nucleatum	494	494	97%	3,00E-173	70.48%	344	WP_098702880.1
	DNA (cytosine-5-)-methyltransferase [Fusobacterium periodonticum D10]	Fusobacterium periodonticum D1	494	494	97%	4,00E-173	70.18%	361	EKA93595.1
	Eco47II family restriction endonuclease [Pseudoleptotrichia goodfellowii]	Pseudoleptotrichia goodfellowii	502	502	97%	1,00E-172	72.29%	600	MBF4804993.1
	DNA cytosine methyltransferase [Campylobacter jejuni]	Campylobacter jejuni	490	490	98%	7,00E-172	70.96%	338	EAI4070807.1
	DNA methyltransferase [Campylobacter jejuni]	Campylobacter jejuni	490	490	98%	1,00E-171	70.96%	347	AXL47160.1
	DNA cytosine methyltransferase [Campylobacter jejuni]	Campylobacter jejuni	489	489	98%	3,00E-171	70.66%	338	WP_002921457.1
13377_J7894_00205	DNA (cytosine-5-)-methyltransferase [[Micrococcus] candicans]	[Micrococcus] candicans	822	822	99%	0.0	65.43%	594	WP_198687347.1
	DNA (cytosine-5-)-methyltransferase [Carnobacterium alterfunditum]	Carnobacterium alterfunditum	805	805	99%	0.0	64.02%	600	WP_081884459.1
	DNA (cytosine-5-)-methyltransferase [Staphylococcus sp. HMSC34C02]	Staphylococcus sp. HMSC34C02	796	796	99%	0.0	63.74%	599	WP_070854712.1
	DNA-methyltransferase Dcm [Mycobacteroides abscessus subsp. abscessus]	Mycobacteroides abscessus subs	795	795	99%	0.0	64.47%	599	SIH36365.1
	DNA (cytosine-5-)-methyltransferase [Staphylococcus haemolyticus]	Staphylococcus haemolyticus	793	793	99%	0.0	63.56%	599	WP_080402264.1
	MULTISPECIES: DNA (cytosine-5-)-methyltransferase [Staphylococcus]	Staphylococcus	793	793	99%	0.0	63.56%	599	WP_070822985.1
	DNA (cytosine-5-)-methyltransferase [Staphylococcus haemolyticus]	Staphylococcus haemolyticus	792	792	99%	0.0	63.39%	599	WP_085060987.1
	DNA (cytosine-5-)-methyltransferase [Staphylococcus haemolyticus]	Staphylococcus haemolyticus	792	792	99%	0.0	63.39%	599	WP_117287751.1
	DNA (cytosine-5-)-methyltransferase [Staphylococcus haemolyticus]	Staphylococcus haemolyticus	790	790	99%	0.0	63.39%	599	WP_080367091.1
	DNA (cytosine-5-)-methyltransferase [Staphylococcus epidermidis]	Staphylococcus epidermidis	789	789	99%	0.0	63.45%	598	WP_115339039.1
4025_J7889_03525	GCATC--recognizing Type II restriction modification system (MmyCIII) adenine DNA methyltransferase subur	synthetic Mycoplasma mycoides J	439	439	97%	1,00E-150	61.94%	362	ADH21784.1
	modification methylase [Lactococcus garvieae]	Lactococcus garvieae	382	382	91%	6,00E-128	56.25%	398	PCS02374.1
	Dam family site-specific DNA-(adenine-N6)-methyltransferase [Streptococcus uberis]	Streptococcus uberis	391	391	92%	3,00E-127	59.23%	709	WP_154631601.1
	Dam family site-specific DNA-(adenine-N6)-methyltransferase [Streptococcus parauberis]	Streptococcus parauberis	385	385	92%	5,00E-125	57.44%	711	WP_139058238.1
	D12 class N6 adenine-specific DNA methyltransferase family protein [Streptococcus pneumoniae NP070]	Streptococcus pneumoniae NP07	375	375	91%	6,00E-125	56.89%	405	EHD56120.1
	D12 class N6 adenine-specific DNA methyltransferase family protein [Streptococcus pneumoniae GA44128]	Streptococcus pneumoniae GA44	375	375	0,91	7E-125	56.89%	406	EHZ51383.1
	DNA adenine methylase [Streptococcus parauberis NCFD 2020]	Streptococcus parauberis NCFD	385	385	0,92	8E-125	57.44%	720	EGE54547.1
	Dam family site-specific DNA-(adenine-N6)-methyltransferase [Vagococcus penaei]	Vagococcus penaei	383	383	0,9	6E-124	56.63%	708	WP_126844542.1
	DNA adenine methylase [Streptococcus pneumoniae]	Streptococcus pneumoniae	376	376	0,91	9E-124	57.19%	509	WP_057607080.1
	Adenine-specific DNA methylase [Enterococcus durans]	Enterococcus durans	372	372	0,94	1E-123	56.65%	398	STQ48463.1

BLASTP on NCBI (https://blast.ncbi.nlm.nih.gov)
Eclude Mollicutes option
Cut-off E-value of <0.001
Filter : Identity between 100% and 50% / Coverage between 100% and 50%

* : CpG methylase, Type III related MTases (MAGa1570, MAGa1580 and MAG1530) and the Type II methylase MTase 13377_964
are not represented in this table as they have no homologs outside Mollicutes class
